# In Silico Design of a Trans-Amplifying RNA-Based Vaccine against SARS-CoV-2 Structural Proteins

**DOI:** 10.1155/2024/3418062

**Published:** 2024-09-30

**Authors:** Fatemeh Nafian, Ghazal Soleymani, Zahra Pourmanouchehri, Mahnaz Kiyanjam, Simin Nafian, Sayed Mohammad Mohammadi, Hanie Jeyroudi, Sharareh Berenji Jalaei, Fatemeh Sabzpoushan

**Affiliations:** ^1^ Department of Medical Laboratory Sciences Faculty of Paramedics Tehran Medical Sciences Islamic Azad University, Tehran, Iran; ^2^ Department of Biological Sciences Virginia Polytechnic Institute and State University, Blacksburg, Virginia, USA; ^3^ Department of Biology Technical University of Kaiserslautern, Kaiserslautern Technical University of Kaiserslautern, Kaiserslautern, Germany; ^4^ Department of Cellular and Molecular Biology Faculty of Advanced Sciences and Technology Tehran Medical Sciences Islamic Azad University, Tehran, Iran; ^5^ Department of Stem Cell and Regenerative Medicine National Institute of Genetic Engineering and Biotechnology (NIGEB), Tehran, Iran; ^6^ Department of Biotechnology Faculty of Converging Sciences and Technologies Science and Research Branch Islamic Azad University, Tehran, Iran; ^7^ Department of Biochemistry Faculty of Converging Sciences and Technologies Science and Research Branch Islamic Azad University, Tehran, Iran

## Abstract

Nucleic acid-based vaccines allow scalable, rapid, and cell-free vaccine production in response to an emerging disease such as the current COVID-19 pandemic. Here, we objected to the design of a multiepitope mRNA vaccine against the structural proteins of SARS-CoV-2. Through an immunoinformatic approach, promising epitopes were predicted for the spike (S), envelope (E), membrane (M), and nucleocapsid (N) proteins. Fragments rich in overlapping epitopes were selected based on binding affinities with HLA classes I and II for the specific presentation to B and T lymphocytes. Two constructs were designed by fusing the fragments in different arrangements via GG linkers. Construct 1 showed better structural properties and interactions with toll-like receptor 2 (TLR-2), TLR-3, and TLR-4 during molecular docking and dynamic simulation. A 50S ribosomal L7/L12 adjuvant was added to its N-terminus to improve stability and immunogenicity. The final RNA sequence was used to design a trans-amplifying RNA (taRNA) vaccine in a split-vector system. It consists of two molecules: a nonreplicating RNA encoding a trans-acting replicase to amplify the second one, a trans-replicon (TR) RNA encoding the vaccine protein. Overall, the immune response simulation detected that activated B and T lymphocytes and increased memory cell formation. Macrophages and dendritic cells proliferated continuously, and IFN-*γ* and cytokines like IL-2 were released highly.

## 1. Introduction

Severe acute respiratory syndrome coronavirus 2 (SARS-CoV-2) emerged in a new pandemic of coronavirus disease 2019 (COVID-19) in China and spread worldwide [1]. So far, 770,085,713 confirmed cases, including 6,956,173 deaths, have been reported to the WHO Coronavirus (COVID-19) Dashboard (last updated: CEST, 30 August 2023). Infections such as pneumonia, diarrhea, enteric symptoms, and renal failure are common. Therefore, the COVID-19 outbreak has significant risks to international health and socioeconomic status. Currently, there are no approved specific antiviral agents against SARS-CoV-2, while some nonspecific drugs are still under investigation, including remdesivir, lopinavir, and ritonavir. In this condition, it needs to control and prevent the spread and severity of COVID-19 through vaccination [2].

To help advance the prevention of COVID-19, RNA-based immunization is seen as an alternative to traditional live or inactivated viral vaccines, subunit vaccines, and DNA vaccines. It can be classified into amplifying mRNA and nonreplicating mRNA (nrRNA). A cap, 5′-untranslated regions (UTR), open reading frame (ORF) containing vaccine antigens, 3′-UTRs, and a poly (A) tail comprise standard nrRNA. In addition, regulating ribosome accessibility, and impacting translational processes are needed. On the other hand, amplifying RNA is divided into self-amplifying RNA (saRNA) and trans-amplifying RNA (taRNA) which contains more trans-acting elements (TSE) required for viral RNA replication [3]. saRNA is a replicon RNA unit containing replicas and transgene for RNA self-replication and antigen expression [4]. However, taRNA is a bipartite system, including one nrRNA encoding the replicas and another trans-replicon RNA (TR-RNA) encoding the vaccine antigen that trans-replicated with the first.

The main structural proteins of SARS-CoV-2 include spike (S), nucleocapsid (N), membrane (M), and envelope (E) proteins [5]. The S glycoprotein binds to the angiotensin-converting enzyme 2 (ACE2) to promote viral entry by a human cell surface receptor [6]. It comprises two subunits (S1 and S2) that form a trimer complex on the viral membrane. The S1 subunit contains a receptor-binding domain (RBD) responsible for binding to host cell receptors. At the same time, the S2 subunit allows membrane fusion and facilitates the viral uncoating process. Two cleavage sites are located between the S1 and S2 subunits (S1/S2) and also in S2. Cleavage is one of the imperative posttranslational alterations related to virulence and pathogenicity [7, 8]. The S protein mainly induces neutralizing antibodies (nAbs) and cellular immune responses. Therefore, it is one of the most promising options for SARS-CoV-2 vaccine development [9].

During the early stages of infection, the N protein is frequently expressed in the host cell to form a ribonucleoprotein complex with the genomic RNA. This complex involves virus entrance into host cells and the following cellular processes. The N protein is highly conserved between different SARS-CoVs, making it an essential component following the S protein. The M protein interacts with N to stabilize the nucleocapsid RNA complex, facilitating viral assembly completion [10]. It helps the S protein to remain in the ER-Golgi intermediate compartment. Furthermore, it has a critical role in morphogenesis, the control of replication, and packing the genomic RNA into viral particles. During assembly and budding, the E protein changes the permeability and morphology of the host cell membrane by cation-selective ion channel activity [11]. E contains a short N-terminal ectodomain (NTD), a triple-spanning transmembrane domain (TMD), and the PDZ-binding domain (PBM) at the C-terminal. It shows the most conserved sequence and similarity with SARS-CoV, approximately 97% [12], and the mutated E protein leads to lower viral titer and immature, inefficient progenies.

The immunopathological response to SARS-CoV-2 infection derives from disease progression, involving dysregulated innate and adaptive immunity and autoimmunity. Viral pathogen-associated molecular patterns (PAMPs) trigger Toll-like receptors (TLRs), notably TLR-4, initiating intracellular signaling cascades that activate nuclear factor-kappa B (NF-*κ*B) and interferon regulatory factors (IRFs) [13–15]. Despite initial suppression by SARS-CoV-2 ORF6 and ORF3b, antiviral IFN-I and pro-inflammatory cytokines eventually escalate, leading to hyperinflammation and organ damage [16, 17]. Combating COVID-19 requires a multifaceted approach, prompting the development of diverse vaccine strategies [18], including inactivated viruses (Sinopharm [19] and Sinovac [20]), protein subunit vaccines (Novavax [21]), nonreplicating viral vectors (AstraZeneca [22], Johnson and Johnson [23], and Sputnik V [24]), DNA-based vaccines (ZyCoV-D [25]), and mRNA-based vaccines (BNT162b2 [26], and Moderna mRNA-1273 [27]). Potential vaccine challenges include epitope recognition failure against new viral mutants, inadequate long-term immunity, and the risk of antibody-dependent enhancement.

This article used an immunoinformatic approach to design a multiepitope taRNA against SARS-CoV-2. At first, the critical B-cell and T-cell epitopes were predicted for all structural proteins, including S, N, M, and E. Subsequently, two multiepitope constructs were designed. Their 3D structures were modeled and evaluated using molecular dynamic simulations. The potential to bind TLRs and generate favorable antiviral responses was confirmed and simulated in silico. Finally, its RNA sequence was engineered as a taRNA vaccine in a split-vector system.

## 2. Materials and Methods

**Figure 1:**
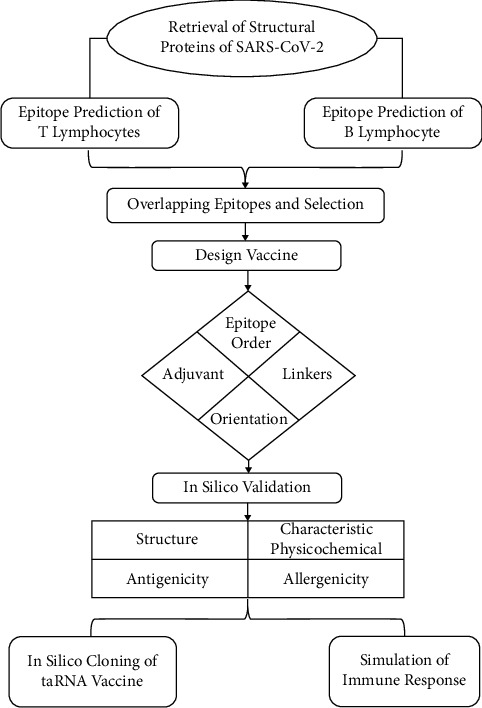
The workflow for designing a trans-amplifying RNA vaccine in silico. Initially, epitopes were predicted and overlapped to construct the vaccine in various orders. Subsequently, an adjuvant was incorporated into the design, and the entire nucleotide sequence was cloned into a split-vector system. Finally, the immune response to the finalized vaccine was simulated.

The flowchart representing the overall procedures of vaccine design is illustrated in Figure 1.

### 2.1. Retrieval of Protein Sequences and Structures

The amino acid sequence was retrieved from the UniProt database (https://www.uniprot.org/). Accession numbers P0DTC2, P0DTC9, P0DTC4, and P0DTC5, are related to S, N, E, and M proteins, respectively [28]. This database presents function, taxonomy, subcellular location, variants, interaction, structure, and domain in proteins.

### 2.2. Epitope Prediction

An ideal vaccine should induce long-lasting immunity that mimics natural immunity by generating cytotoxic T-cell (CTL) and helper T-cell (HTL) epitopes. CTL epitopes help build long-lasting immunity that can eliminate the circulating virus and the virus-infected cells. HTL epitopes help develop protective CD8+ T-cell memory and produce antibodies by B-cells to generate cellular and humoral responses. Therefore, an efficient vaccine candidate should carry HTL and CTL receptor-specific epitopes [29].

Long CTL epitopes were predicted using the NetMHCIIPan 4.1 EL method [30] and also TepiTool [31], according to the recommended IEDB (Immune Epitope Database and Analysis Resource) official manual (https://www.iedb.org) with AUC values greater than 0.9 [32, 33]. The performance of predictions is quantified using the area under the curve (AUC), where 0.5 signifies random prediction and 1.0 signifies perfect prediction. They can be recognized by HLA class I (HLA-I). On the other hand, long HTL binding epitopes were predicted using recommended IEDB 2.22 which combines the NetMHCIIPan 3.2 method with the consensus method (SMM-align, NN-align, and CombLib/Sturniolo) to be recognized by HLA class II (HLA-II) with AUC of 0.87 [34]. The Tepitool method was also used to confirm predicted long HTL epitopes related to HLA-II. Strong binders were screened based on their high predicted scores by both methods. In the end, epitopes with an affinity for multiple HLA alleles were selected as overlapping epitopes of HLA-I and HLA-II, which can induce a relatively immune response in the host cell.

B-cell receptors can induce an immune response by recognizing B-cell epitopes and producing antibodies. The presence of these specific vaccine epitopes is vital in helping to generate an effective immune response [35]. The online tools in the IEDB were used to analyze the conserved regions of the candidate epitopes (https://tools.iedb.org/bcell). Continuous B-cell epitopes were predicted using BepiPred-2.0 (0.68 AUC), Karplus-Schulz (0.59 AUC), and Chou-Fasman (0.64 AUC) servers with thresholds of 0.6, 1.1, and 1.2, respectively [36–38]. The accuracy of B-cell epitope prediction tools is generally rather poor, having AUC values ranging from 0.6 to 0.7 [39]. A combination of these parameters was evaluated to improve the accuracy of B-cell epitope prediction. The presence of epitopes on the surface of proteins was also confirmed using Karplus and Chou_Fasman servers' thresholds 1.1 and 1.2, respectively. In addition, discontinuous B-cell epitopes were predicted using ElliPro (https://tools.iedb.org/ellipro/), based on 3D structures of S, M, N, and E proteins.

### 2.3. Identification of Overlapping Epitopes

The Molecular Evolutionary Genetics Analysis (MEGA) software was used to align the selected epitopes and identify the overlapping epitopes [40]. First, the adjacent HTL and CTL epitopes as well as continuous B-cell epitopes were bridged to form candidate epitope-rich fragments with lengths no more than 30 aa. Class I immunogenicity was considered for fragments with a score above 0 (https://tools.iedb.org/immunogenicity/) [41]. The physicochemical properties were predicted, such as a grand average of hydropathicity (GRAVY), instability index, theoretical isoelectric point (pI), and aliphatic index, by using the ProtParam-ExPASy server (https://web.expasy.org/protparam/) [42].

### 2.4. Interaction Analysis of Epitope-Rich Fragments and Specific HLA Alleles

The binding affinity was measured between epitope-rich fragments (overlapping regions) and the HLA-I and HLA-II. First of all, the three-dimensional structures of fragments were predicted using PEP-FOLD3 through a de novo method from the amino acid sequences (https://bioserv.rpbs.univ-paris-diderot.fr/services/PEP-FOLD3/) [43]. Then, each predicted 3D structure was separately docked as a ligand, with the most prevalent HLA alleles in the human population as receptors. The structures of HLA-A^∗^02:01 and HLA-DRB1^∗^01:01-DRA ^∗^01:01 were retrieved from the Protein Data Bank (PDB) with IDs 1QEW and 1YMM, respectively (https://www.rcsb.org) [44]. AutoDock4.0 predicted bound conformations by the Lamarckian Genetic Algorithm in a free energy force field [45].

### 2.5. Modelling and Comparative Analysis of Different Vaccine Constructs

According to their physicochemical properties, two different arrangements of fragments were connected by GG linkers to create two multiepitope constructs. Then, constructed 3D structures were modeled using I-TASSER (https://zhanggroup.org/I-TASSER/) [46]. Depending on the protein sequences, I-TASSER modeled four anticipated candidates in the PDB arranging and reported a brief clarification of the RMSD, TM-core, and C-score. After that, molecular dynamics (MD) simulation was performed to find a more stable structure in the OPLS-AA force field [47]. The fundamental steps for the whole procedure included topology generation, solvation in a particular ion concentration, energy minimization through 50,000 steps of 1 fs, heating to 300 K from an initial temperature of 100 K, and equilibration for a total time of 100 ps. We calculated the radius of gyration (RGYR), RMSD, and RMSF (root-mean-square fluctuation) to analyze the results, concerning energy-minimized structures using the GROMACS algorithm.

The more stable construct was docked with human toll-like receptors 2 (TLR-2), 3 (TLR-3), and 4 (TLR-4) by the ClusPro2.0. Server (https://cluspro.bu.edu/home.php) [48]. Construct-TLR complexes were visualized using the PyMOL software (https://pymol.org/) [49]. A natural adjuvant was also attached to the N-terminus of the selected construct using an EAAAK linker. It is the 50S ribosomal L7/L12 protein from *Mycobacterium tuberculosis* (Rv0652); with the accession number of P9WHE3 in UniProt. The 3D structure of the final vaccine construct was predicted using the I-TASSER server and refined by locPREFMD (https://feig.bch.msu.edu/web/services/locprefmd), a local Protein structure REFinement via Molecular Dynamics [50]. The quality model was evaluated based on the Ramachandran map plotted in SAVESv6.0 (https://saves.mbi.ucla.edu/).

### 2.6. Antigenicity and Allergenicity Prediction

The protective antigenicity of the vaccine construct can be predicted through the alignment-independent approach using the physicochemical properties [51]. Therefore, we used VaxiJenv.2.0 (https://www.ddg-pharmfac.net/vaxijen) and ANTIGENPro (https://scratch.proteomics.ics.uci.edu/) to predict antigenicity with a threshold of 0.3. Furthermore, the AllerTOPv.2.0 server was also applied to predict allergenicity in silico (https://www.ddg-pharmfac.net/AllerTOP/) [52].

### 2.7. Population Coverage Analysis for Selected Epitopes

A population coverage analysis was conducted to ensure the designed vaccine construct elicits a robust immune response across diverse global populations. This analysis was performed using the IEDB Population Coverage Tool, which assesses the distribution of selected T-helper and cytotoxic T-cell epitopes across various MHC-restricted alleles. Nine epitopes were analyzed for population coverage, incorporating both Class-I and Class-II MHC molecules. The selected epitopes, along with their corresponding MHC-restricted alleles, were entered into the tool, and the results were computed [53].

### 2.8. Engineering Sequence taRNA Vaccine in a Split-Vector System

A split-vector system was engineered in two RNAs. The first was typically an nr-RNA that encoded a replicase to amplify the second, TR-RNA encoding the antigen. Both contained the basic elements of mRNA (a cap, 5′ UTR, 3′ UTR, and poly(A) tail). However, nr-RNA had a long ORF encoding four viral nonstructural proteins (nsPs), nsP1 to nsP4, as an NTPase, helicase, protease, and RNA-dependent RNA polymerase (RDRP), respectively. Instead, TR-RNA carried the mRNA sequence of the vaccine candidate containing Construct 1 and adjuvant under the control of a subgenomic promoter (SGP).

### 2.9. In Silico Evaluation of Immune Response

To evaluate the immune response profile of the multiepitope vaccine, in silico immune simulations were performed by the C-ImmSim online server (https://kraken.iac.rm.cnr.it/C-IMMSIM/) [54]. It applies the machine learning method and Celada-Seiden model to evaluate humoral and cellular responses against an injected vaccine in silico. Clinically, the vaccine candidate was injected for eight hours. The parameters of the simulation were default with a volume of 10, random seeds of 12,345, and 1000 steps [55].

## 3. Results

### 3.1. Characterization of Overlapping Epitopes

It is a crucial step in the immune responses that epitopes stimulate CTLs and HTLs or are trapped by B-cells to release soluble antibodies. Long CTL epitopes were predicted using NetMHCPan 4.1 EL and TepiTool. The protein sequences were split into small fragments with a length of 9-10 amino acids. Their binding affinity was calculated with the frequently HLA-I alleles (HLA-A ^∗^01:01, HLA-A ^∗^02:01, and HLA-B ^∗^07:02 with allele-specific affinity cutoff (IC50) 884, 255, and 687 nM, respectively) [56].  Table 1 shows only those with the top 1% binding affinity in column 3, according to the percentile rank of ≤1 and < 0.3 in NetMHCIIPan 4.1 EL and TepiTool, respectively. Long HTL binding epitopes were also predicted using the recommended IEDB 2.22 and TepiTool methods to be recognized by the 7-alleles HLA-II reference set with a percentile rank of ≤2 and <0.3, respectively. The selected HLA-II alleles included HLA-DRB1 ^∗^01:01, HLA-DRB1 ^∗^03:01, HLA-DRB1 ^∗^04:01, HLA-DRB1 ^∗^04:05, HLA-DRB1 ^∗^07:01, HLA-DRB1 ^∗^08:02, HLA-DRB1 ^∗^09:01, HLA-DRB1 ^∗^11:01, HLA-DRB1 ^∗^12:01, HLA-DRB1 ^∗^13:02, HLA-DRB1 ^∗^15:01, HLA-DRB3 ^∗^01:01, HLA-DRB3 ^∗^02:02, HLA-DRB4 ^∗^01:01, HLA-DRB5 ^∗^01:01, HLA-DQA1 ^∗^05:01/DQB1 ^∗^02:01, HLA-DQA1 ^∗^05:01/DQB1 ^∗^03:01, HLA-DQA1 ^∗^03:01/DQB1 ^∗^03:02, HLA-DQA1 ^∗^04:01/DQB1 ^∗^04:02, HLA-DQA1 ^∗^01:01/DQB1 ^∗^05:01, HLA-DQA1 ^∗^01:02/DQB1 ^∗^06:02, HLA-DPA1 ^∗^02:01/DPB1 ^∗^01:01, HLA-DPA1 ^∗^01:03/DPB1 ^∗^02:01, HLA-DPA1 ^∗^01:03/DPB1 ^∗^04:01, HLA-DPA1 ^∗^03:01/DPB1 ^∗^04:02, HLA-DPA1 ^∗^02:01/DPB1 ^∗^05:01, and HLA-DPA1 ^∗^02:01/DPB1 ^∗^14:01. Peptides predicted to bind to these alleles were selected by the IEDB using a consensus percentile rank of the top 10% and an IC50 cutoff of 1000 nM. Strong binders with a length of 15 amino acids are shown in columns 4 and 5 in Table 1, which were detected by TepiTool and NetMHCIIPan 4.1 EL, respectively.

BepiPred 2.0, Karplus-Schulz, and Chou-Fasman, also predicted continuous B-cell epitopes with thresholds of 0.6, 1.1, and 1.2, respectively (column 6, Table 1). This setup detected consensus B-cell epitopes only in S and N proteins, stimulating antibody production. The ElliPro analysis server utilizes the PDB file of specific proteins for discontinuous B-cell epitope prediction, rather than amino acid sequences, as input data and gives an AUC value of 0.73 [57]. Therefore, protein files from the PDB named 6X29, 7VGR, 6 M3M, and 7 K3G were utilized to evaluate the different epitopes of S, M, N, and E, respectively. Discontinuous epitopes with scores above the minimum threshold of 0.5 were selected and are presented in Supplementary Tables 1, 2, 3, and 4.

Finally, high-binding epitopes to all HLA-I and HLA-II alleles and B-cells were considered consensus epitopes (overlapping epitopes, Table 1, column 2). Four epitope-rich fragments were selected from the spike protein in positions 51–75, 812–835, 336–359, and 451–477, with sequences of TQDLFLPFFSNVTWFHAIHVSGTNG (named S1), PSKRSFIEDLLFNKVTLADAGFIK (S2), CPFGEVFNATRFASVYAWNRKRISN (S3), and YLYRLFRKSNLKPFERDISTEIYQAGST (S4), respectively. Another selected fragment was MADSNGTITVEELKKLLEQW (M) from the extracellular region of membrane protein, in amino acids 1 to 20. Next overlapping epitope was related to the nucleocapsid protein with the sequence of PANNAAIVLQLPQGTTLPKGFYAE (N2), according to 151 to 174 amino acids. This position is located at the C-terminal of the RNA binding domain (RBD). The selected fragment of the envelope protein was MYSFVSEETGTLIVNSV (E), in amino acids 1–17. Fragments indicated a high score of Class I immunogenicity (Table 2, column 3). The physicochemical properties included the aliphatic index, GRAVY, theoretical isoelectric point (pI), molecular weight, and instability index. The results are revealed in detail in Table 2. An instability index smaller than 40 shows a stable protein. The GRAVY value proves the sum of the amino acids' hydropathy values, divided by the number of residues. The aliphatic index is a positive factor in increasing thermostability based on the calculation of the aliphatic amino acids.

**Figure 2:**
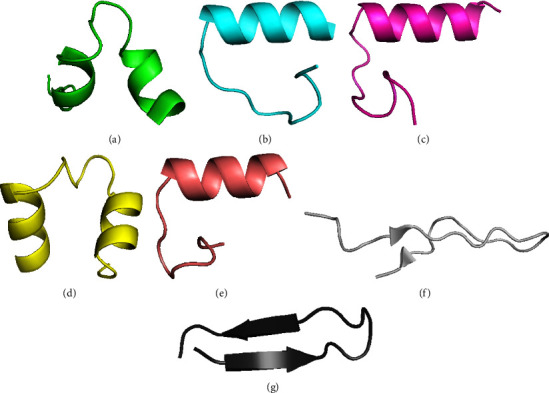
The 3D structures of epitope-rich fragments that were computationally modeled by the PEP-FOLD3 server. The best-predicted models had the lowest potential energy presented for Fragment S1 (a), Fragment S2 (b), Fragment S3 (c), Fragment S4 (d), Fragment M (e), Fragment N2 (f), and Fragment E (g).

The 3D structures of epitope-rich fragments were predicted based on their consensus sequences using the PEP-FOLD3 server.  Figure 2 shows the best of them with the lowest coarse-grained energy of PEP-FOLD3 (sOPEP).

Molecular docking calculated binding energies between each 3D structure and the most prevalent HLA alleles. AutoDock4 measures binding energy in a comprehensive thermodynamic force field that evaluates hydrogen bonds, buried surface area, allosteric effects, and geometric angles. The results are shown in Table 3. The names of the ligand and receptor are given in columns 1 and 2, respectively. The intramolecular energy in column 3 is the interaction of atoms within the ligand or receptor for the transition from the unbound states to the bound conformation. In column 4, the intermolecular energy is the sum of the vdv_hb_desolv energy and the electrostatic energy in the bound conformation.

### 3.2. Designing Multiepitope Vaccine Constructs

**Figure 3:**
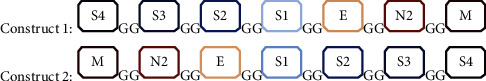
Schematic diagram of differently designed constructs. Construct 1 was designed by arranging fragments as S4, S3, S2, S1, E, N2, and M with the help of GG linkers from the N to the C-terminus of the protein. Construct 2 contained the arrangements of M, N2, E, S1, S2, S3, and S4, respectively.

Two distinct arrangements of the chosen fragments were designed based on their physicochemical properties, like GRAVY and hydropathicity (Figure 3). Low hydropathic fragments were S4, S3, and M, with GRAVY values of −0.739, −0.348, and −0.540, respectively. Due to their hydrophilic properties, they were located near two termini of a designed construct. On the other hand, high hydropathic fragments were S1, S2, N2, and E with GRAVY values of 0.244, 0.087, and 0.565, respectively. They were placed in the middle of a structure according to their hydrophobic properties. Fragments were linked together using GG linkers.

The 3D structure of each construct was predicted using I-TASSER based on the top ten threading templates with defined structures. I-TASSER recommends the first model with the highest confidence score (C-score) and topological similarity to a template (TM-score) that has the lowest root-mean-square deviation (RMSD) [58]. For modeling Construct 1, I-TASSER used ten templates for prediction with PDB IDs 3jclA, 7sblA, 1ssk, 6jx7, 5i08A, 6 nb3A, 5i08A, 7p1hA, 7dwyA, and 1sskA. It had the highest C-score of −1.21, TM-score of 0.56 ± 0.15, and RMSD of 7.8 ± 4.4 Å. For construct 2, I-TASSER predicted 3D structure based on 10 threading templates including 6u7h, 7sxsA, 6 b7n, 7rkvA, 6u7hA, 7a93A, 7qo7A, 7sblA, 6b7nA, and 7czpA. The best 3D structure had the highest C-score of −4.48, TM-score of 0.25 ± 0.07, and RMSD of 16.1 ± 3.1 Å.

### 3.3. Comparative Analysis of Different Vaccine Constructs

**Figure 4:**
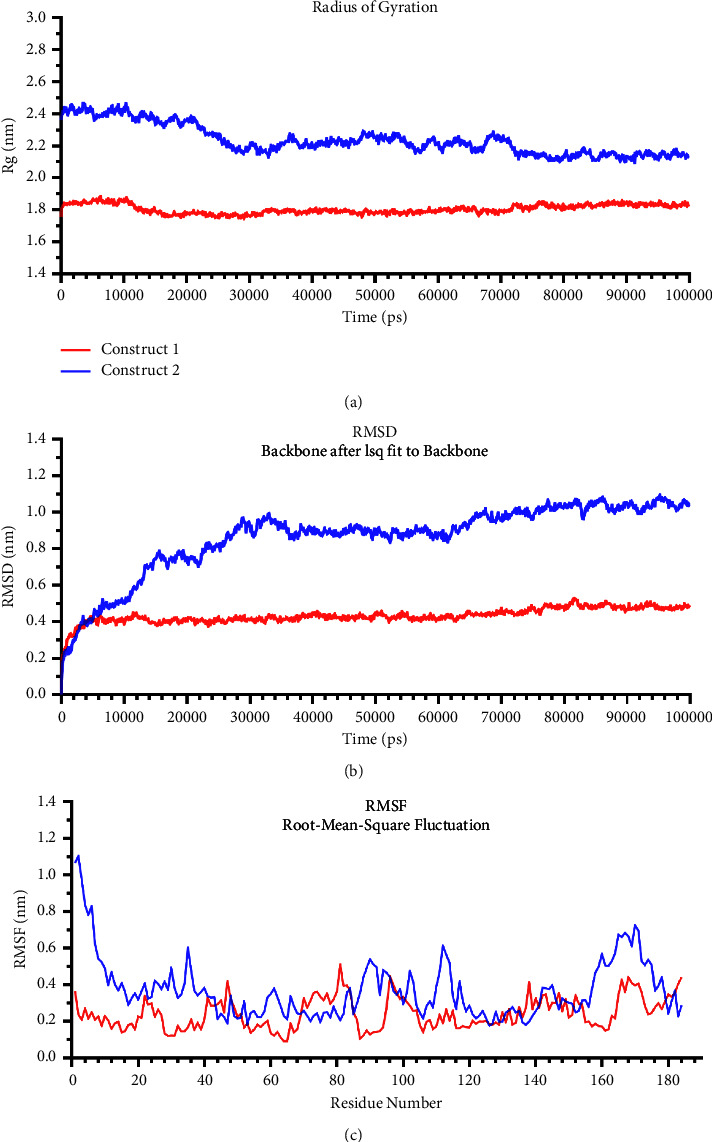
Conformational changes in Construct 1 (red) and 2 (blue) predicted structures. (a) The radius of gyration (Rg) of constructs during 100000 ps (100 ns) time of molecular dynamics (MD). (b) RMSD plot of the predicted structure of constructs during 100000 ps (100 ns) MD. (c) RMSF plot for fluctuation of every residue in each construct.

Two constructs were taken to molecular dynamics using the OPLS force field to investigate the structural stability based on the radius of gyration (Rg), RMSD, and RMSF analyses. The flexibility of conformation was quantitatively checked by analysis of the Rg curve for their structure (Figure 4(a)). Construct 1 showed the most stable Rg values with an average of about 1.804 nm. In contrast, the corresponding value for another construct had nonstop decreased during the simulation's time. Figure 3 also shows RMSD variations of protein backbone atomic coordinates during 100000 ps (100 ns) time of MD simulation (Figure 4(b)). In Construct 1, RMSD values were balanced after 10000 ps, maintaining average fluctuations on 0.531 Å. On the other hand, the mean deviation of atomic positions in Construct 2 was more, about 0.864 Å. Its RMSD values continuously increased to the end, up to 1.043 Å. The fluctuations were also compared for every residue of constructs in which RMSF values of Construct 1 were less than 0.5 nm compared to Construct 2 (>1 nm) (Figure 4(c)). It suggested that Construct 1 has achieved an equilibrium state and structural stability against another. It is selected to incorporate into the final vaccine design.

### 3.4. Molecular Docking of the Selected Construct with TLR-2, TLR-3, and TLR-4 Receptors

**Figure 5:**
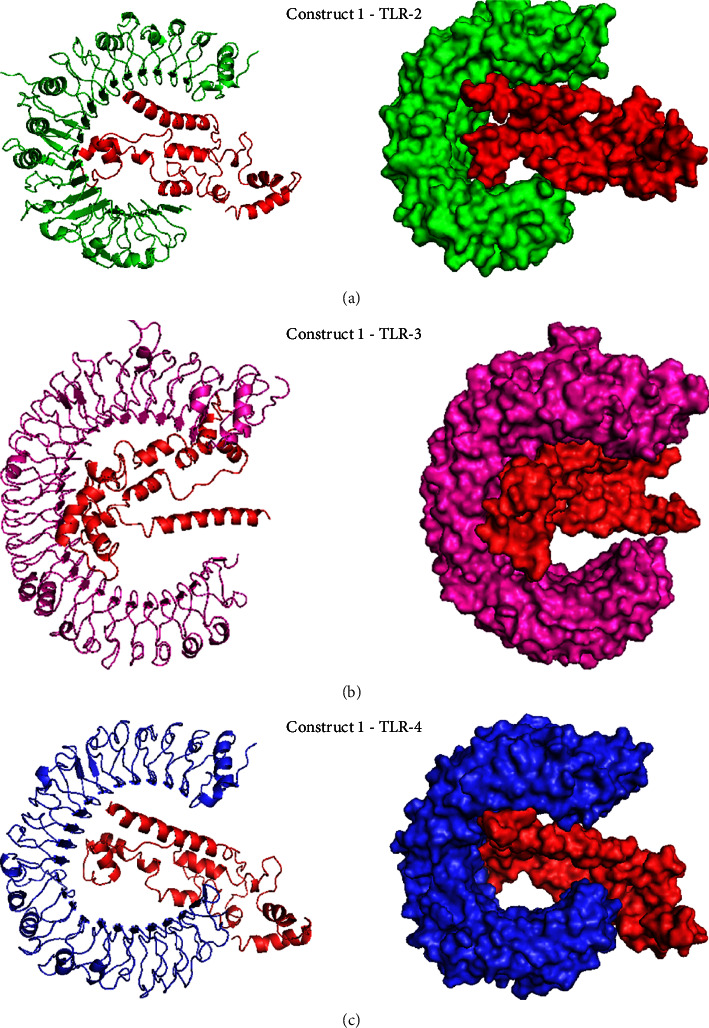
The 3D structure of the best clusters with the lowest energy. (a) The cluster between Construct 1 (red) and TLR-2 (green), (b) Construct 1 and TLR-3 (purple), (c) Construct 1 and TLR-4 (blue) in the cartoon shapes (right) and surface shapes (left).

The ClusPro2.0 server docked vaccine construct as a ligand with each receptor, TLR-2 (PDB ID: 2Z7X), TLR-3 (PDB ID: 2 A0Z), and TLR-4 (PDB ID: 2Z63). It samples billions of conformations and performs pairwise RMSD energy minimization to deliver cluster ratings based on rigid docking. The lowest energy scores were −1237.4, −1337.5, and −1324.7 for the best clusters of TLR-2, TLR-3, and TLR-4, respectively. These complexes were visualized using PyMOL in Figure 5.

**Figure 6:**
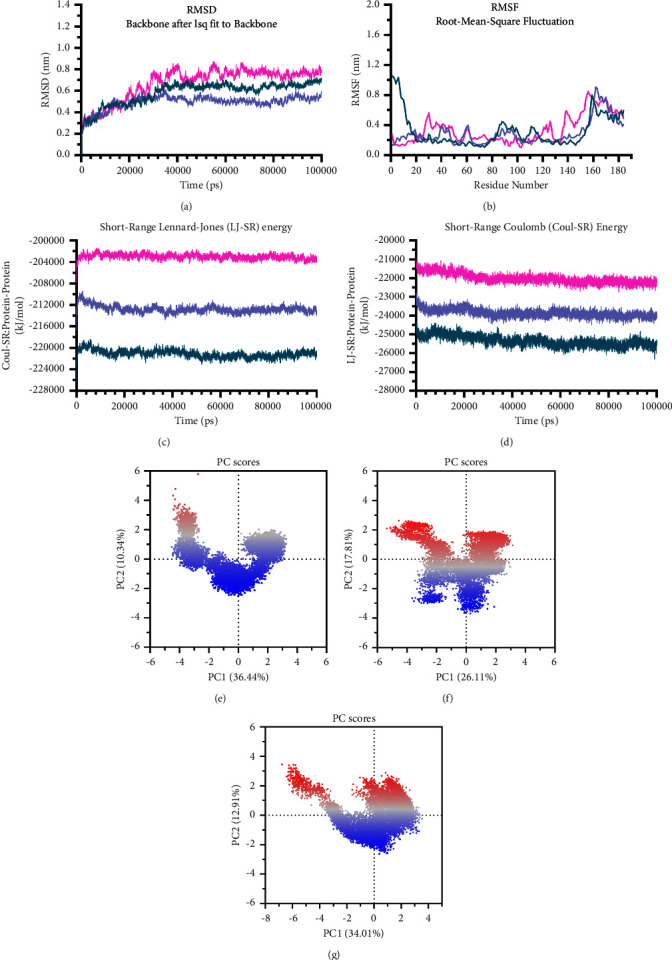
Simulation analysis of Construct 1 in complexing with TLR-2 (pink), TLR-3 (deep teal), and TLR-4 (light blue). (a) RMSD plot of Construct 1 in complex with TLR-2, TLR-3, and TLR-4 for 100 ns of simulation. (b) RMSF values of Construct 1 residues in complex with TLR-2, TLR-3, and TLR-4. (c, d) Changes in interaction energy between Construct 1 in complex with each TLR during the simulation time. (e, f, g) Principal component analysis by clusters of Construct 1 from internal modes, in interacting with TLR-2 (e), TLR-3 (f), and TLR-4 (g) dynamics. During the MD simulation, PCA resulting trajectory frames change from red to white to blue conformation across the top two PC1 vs PC2 spaces.

After docking, 100 ns molecular dynamics were performed on Construct 1 in complex with each of the TLR-2, TLR-3, and TLR-4 receptors to analyze the binding stability. The binding stability of Construct 1 was evaluated as a ligand in the cluster with TLR-2, TLR-3, and TLR-4 based on RMSD and RMSF analyses. The RMSD of the vaccine-TLR-4 cluster showed stable binding interaction with small deviations from its initial position maintained between 0.4 and 0.5 nm (Figure 6(a)). The atomic coordinates of clusters of vaccine with TLR-2 and TLR-3 receptors displayed greater deviation from its initial structure throughout the simulation maintained around 0.7 and 0.6 nm, respectively. However, all RMSD values of the three clusters were less than 1 nm, detecting that vaccine bindings to each TLR are relatively stable. The fluctuations were also compared for every residue of Construct 1 in complex with TLR-2, TLR-3, or TLR-4 in the RMSF plot (Figure 6(b)). Residue-based fluctuations measure how far the atomic location of the vaccine has deviated from its mean structure and how dynamic the ligand-TLR interactions are. In this analysis, the RMSF values of Construct 1 in all complexes projected were less than 1 nm. The most fluctuation was detected at the N- and C-terminus and more stable residues were related to the middle of Construct 1. Additionally, protein-protein interactions were assessed during the simulation using obtained negative values of the short-range Coulomb (Coul-SR) and short-range Lennard-Jones (LJ-SR) energies. The LJ-SR: protein-protein values of interaction energy of Construct 1 with TLR-2, TLR-3, and TLR-4 are −22016.9 kJ/mol, −25343.5 kJ/mol, and −23860.4 kJ/mol, respectively (Figure 6(c)). The average Coul-SR: protein-protein values of interaction energy of Construct 1 with TLR-2, TLR-3, and TLR-4 were −202985.2 kJ/mol, −221106.9 kJ/mol, and −212754.7 kJ/mol, respectively (Figure 6(d)). The average results of protein-protein interaction energy were similar for Construct 1 in all complexes. However, Construct 1 formed thermodynamically firm bonds with TLR-3 better than both TLR-2 and TLR-4. This finding agreed with the principal component analysis (PCA) results that detected Construct 1's conformational changes induced by each TLR binding and overall collective motions of MD trajectories. According to Figure 6, graphs of PC1 vs PC2 depicted these changes in all clusters when Construct 1 was complexed with TLR-2 (e), TLR-3 (f), and TLR-4 (g). Notably, the red region exhibited the most significant movements, the gray region intermediate, and the blue region had the least flexible movements. PC analysis indicated that PC1 clusters retained the highest variability (36.44%, 26.11%, and 34.01% in TLR-2, TLR-3, and TLR-4 complexes, respectively), followed by PC2 (10.34%, 17.81%, and 12.91%), and PC3 showing minimal variability (9.14%, 7.88%, 8.25% values that were not displayed in the figure). This suggests highly stabilized Construct 1-TLR bindings and compact structures.

**Figure 7:**
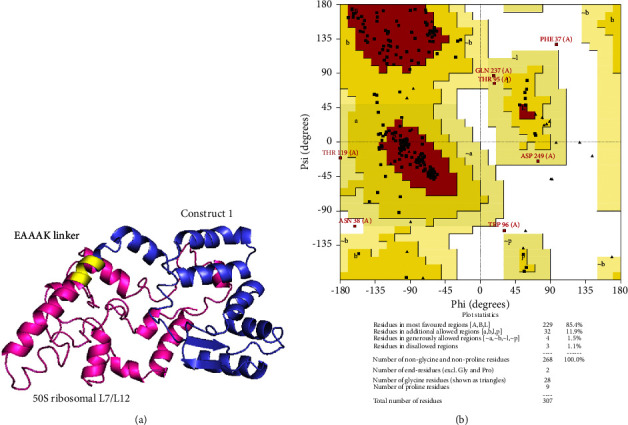
Evaluation of the refined model of the vaccine construct. (a) The final vaccine contains Construct 1 and 50S ribosomal L7/L12 adjuvant. (b) The vaccine structure was validated by Ramachandran map analysis.

Finally, Construct 1 illustrated suitable properties to be used in the design of the final multiepitope vaccine with the help of 50S ribosomal L7/L12 adjuvant (130 aa). It was attached to the N-terminus of Construct 1 using the EAAAK linker, following a similar approach to other studies [59]. The 3D structure of the vaccine candidate was modeled by I-TASSER and refined by locPREFMD (Figure 7(a)). According to the Ramachandran map, the percent of amino acids in favored regions was 85.4%, in additional allowed regions 11.9%, in generously allowed regions 1.5%, and in outlier regions, 1.1% (Figure 7(b)).

The VaxiJenv2.0 and ANTIGENPro servers determined that the proposed vaccine is a probable antigen. The AllerTopv2.0 predicted that the vaccine probably is not an allergen. The physicochemical properties were measured using the ProtParam-Expasy server, summarized in Table 4.

The distribution and expression of HLA alleles vary significantly across different regions and ethnicities worldwide. Assessing this variation is crucial for successful vaccine development to ensure broad efficacy across diverse populations. This study evaluated the global effectiveness of the vaccine designed against SARS-CoV-2. Our analysis revealed that the selected epitopes provide combined population coverage of 94.46% across both HLA classes (Table 5). Specifically, the epitopes achieved coverage rates of 69.53% for Class I and 81.81% for Class II (see Supplementary Figure 1). These findings suggest that the designed multiepitope vaccine has the potential to effectively combat SARS-CoV-2 on a global scale.

### 3.5. Sequence-Engineered taRNA Vaccine in a Split-Vector System

**Figure 8:**
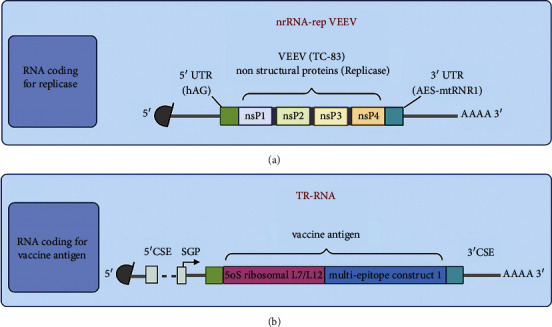
Schematic illustration of trans-amplifying (ta) RNA vaccine in a split-vector system. (a) VEEV replicase ORF is shown in a nonreplicating mRNA (nrRNA) that is flanked by alpha-globin 5′ UTR and AES-mtRNR1 3′ UTR. (b) mRNA sequence of multi-epitope vaccine is shown on a TR-RNA that is flanked by VEEV conserved sequence elements (5′ CSE and 3′ CSE) and subgenomic promotor (SGP).

A nrRNA molecule was designed based on a Venezuelan equine encephalitis virus (VEEV) genome backbone (Figure 8). This genome is an 11.5 kb-long positive RNA that contains a 5′ cap and 3′ poly(A)-tail, similar to the structure of eukaryotic mRNAs. Its ORF1 encodes nsP1, nsP2, nsP3, and nsP4 required to develop a trans-acting replicase. We used the human alpha-globin 5′ UTR was added upstream to increase translational activity. A fusion of motifs derived from amino-terminal enhancer of split (AES) mRNA and mitochondrially encoded 12S rRNA (mtRNR1) was located downstream as a 3′ UTR. It has been demonstrated that the AES-mtRNR1 3′ UTR has the lowest number of predicted binding sites for miRNAs and the highest total hybridization energies [60]. The 5′ alpha globin and 3′ AES-mtRNR1 UTRs are utilized in the Pfizer-BioNTech vaccine to enhance mRNA stability and translation efficiency [61]. Therefore, RNA stability can be improved by it with a segmented 70-nt poly(A) tail. Except for the replication apparatus, TR-RNA was designed similarly by using a 51-nt conserved sequence element (CSE) of the VEEV nsP1 gene at the 5′ end of the mRNA vaccine sequence and 3′ CSE, and the poly(A) tail at the 3′ end. The CSE elements ensured a specific RNA amplification by the replicase.

Notably, the vaccine design excludes a signal peptide, which is typically used in full-length protein vaccines to direct nascent proteins to the secretory pathway [62]. In the context of our multiepitope vaccine, the selected epitopes are intended for direct MHC class I and class II presentation, bypassing the need for secretion and extracellular processing. This omission streamlines the vaccine construct, focusing on the immunogenicity of the targeted epitopes and minimizing potential complexities associated with unnecessary protein trafficking. While this design choice is aligned with the specific goals of our vaccine, the potential role of signal peptides in enhancing MHC presentation and overall vaccine efficacy is acknowledged, leaving room for future exploration in refining and optimizing multiepitope vaccine strategies.

### 3.6. In Silico Evaluation of Immune Response

**Figure 9:**
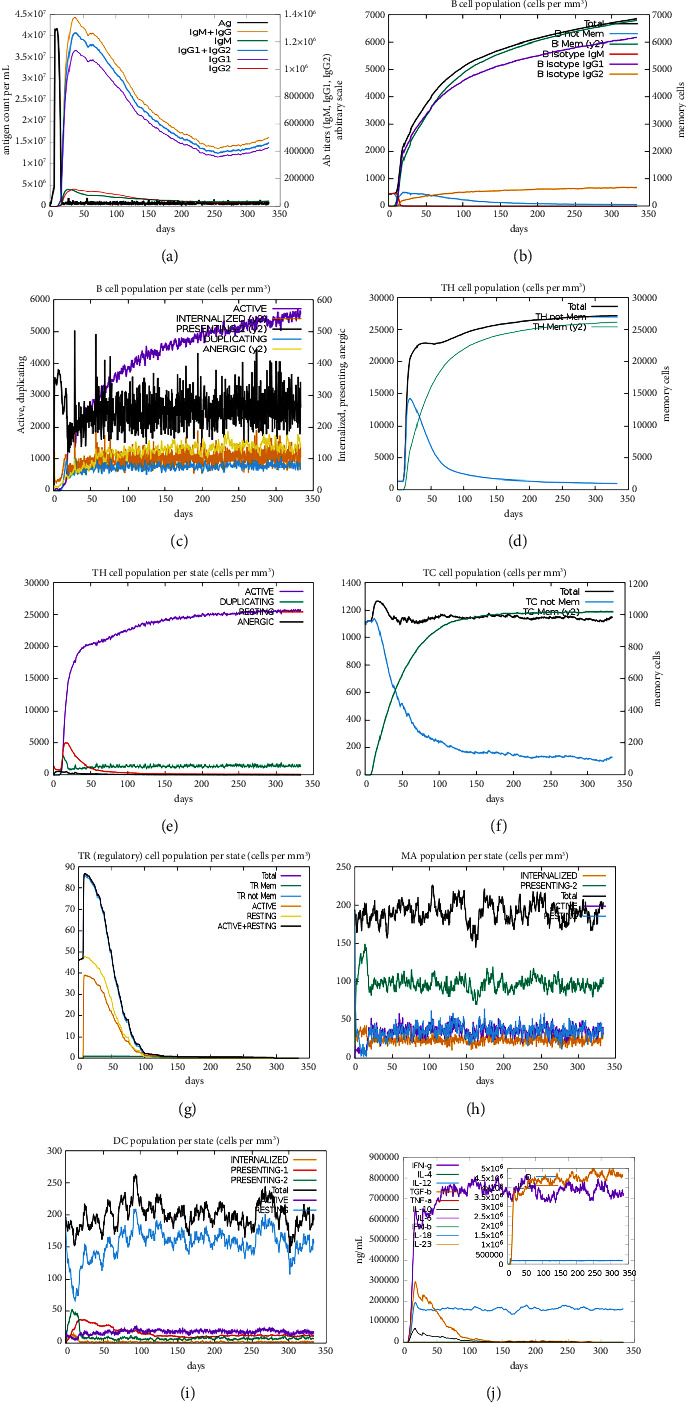
The immune simulation results in silico by injecting the vaccine. (a and b) Immunoglobulins (IgM, IgG1, or IgG2) and B lymphocytes production; (c) memory B-cells formation; (d and e) helper T lymphocytes (HTL) production; (f) cytotoxic T lymphocytes (CTL) production; (g) T regulatory cells reduction; (h) macrophages proliferation; (i) dendritic cells proliferation; and (j) different cytokine levels.

T- and B-cells' activity increased after simulating the immune system in silico. The primary response was indicated by increasing IgM in the beginning. Following, the levels of IgM + IgG, IgG1 + IgG2, and IgG2 were increased despite decreased antigen concentrations (Figure 9(a)). The high activation of B-cells and formation of memory cells determined a long-lasting and effective immune response (Figures 9(b) and 9(c)). Likewise, the development of HTLs, CTLs, and memory cells was observed (Figures 9(d), 9(e), 9(f)). During exposure to antigen, T regulatory cells were decreased (Figure 9(g)), while macrophages and dendritic cells were continuously proliferated (Figures 9(h) and 9(i)). IFN-*γ* and cytokines like IL-2 showed higher levels (Figure 9(j)). CTL and HTL epitopes could enhance IFN-*γ* inducing capability to activate macrophages and NKCs in both the native and specific immune responses. Eventually, the vaccine injection demonstrated the induction of a good antiviral immune response.

## 4. Discussion

A prophylactic vaccine is needed against high-infective and transmission-speed viruses like SARS-CoV-2 [63]. Immunoinformatics has frequently been used to design many advanced multiepitope vaccines [64–68]. The genome and proteome of SARS-CoV-2 have been recently characterized [69–71]. The structural proteins in coronavirus (E, N, M, and S) are essential to produce a complete virus [72]. Well-known that S is a peripheral protein with a central role in the particular virion binding to the receptor on the host cell surface. Thus, the S protein has been one of the main targets for B-cell epitope screening and vaccine development [73, 74]. B and CTL epitopes have been particularly predicted for the spike protein [75]. Several multiepitope vaccines based on this protein have been developed to trigger T lymphocytes (CTLs and HTLs) [76–78]. These vaccines could induce neutralizing antibodies against the spike protein to block SARS-CoV-2 binding and fusion. However, other proteins also are ideal candidates for designing vaccines. Studies have also detected antibodies against Nucleocapsid and Membrane proteins in patients' serum [79]. Also, the E protein is a vitally important envelope component in the virus's assembly, release, and virulence phases [80]. Several proposed vaccines against E, N, M, and ORF3a proteins [81, 82].

In the current study, all structural proteins were used for multiepitope vaccine design by an immunoinformatics approach. In the beginning, overlapping epitopes were determined to selectively elicit all B-cells, HTLs (CD4 +), and CTLs (CD8 +) in both humoral and cellular immunity. Although B-cell induction is chosen for vaccine design, T lymphocytes elicit a strong immunoreaction against viral infections. We predicted HTL and CTL epitopes to induce a more effective cellular immune response through HLA-I and HLA-II antigen recognition. CTL epitopes were predicted by choosing HLA-A ^∗^01:01, HLA-A ^∗^02:01, and HLA-B ^∗^07:02 alleles, which cover ∼95% of the world's people. HTL epitopes were detected by the 7-alleles HLA-II reference set, which can produce IFN-*γ* and maintain CTL responses. Consensus fragments were found in all S (S1, S2, S3, and S4), M, N, and E proteins. They were rich in all B-cell, CTL, and HTL epitopes and anticipated formulating a more effective vaccine against the virus. Their accessibility was also confirmed structurally. During the molecular docking, the clusters of proposed fragments and HLA-A ^∗^02:01 or HLA-DR show a high binding affinity.

Two constructs were designed by altering the order of splicing fragments together to select a better arrangement. After structure predictions by I-TASSER, Construct 1 showed more stability through molecular dynamics. Before the addition of the adjuvant, Construct 1's binding affinity was evaluated by docking with various immune receptors associated with viral infections, such as TLR-2, TLR-3, and TLR-4, using ClusPro2.0. TLRs, expressed either on cell surfaces (TLR-2 and TLR-4) or intracellularly (TLR-3, occasionally TLR-4), play distinct roles in recognizing viral components. Binding to TLR-2/TLR-4 recruits an adaptor protein, MyD88 (myeloid differentiation primary-response protein 88). The MyD88-dependent pathway initiates downstream signaling cascades that lead to the production of various proinflammatory cytokines, ultimately activating both Th1 and Th2 immune responses. Conversely, binding to TLR-3/TLR-4 recruits another adaptor protein, TRIF (TIR domain-containing adaptor protein inducing interferon-*β*). The TRIF-dependent pathway initiates a distinct signaling cascade leading to the production of type-I interferons, which are key molecules in antiviral immune responses [83]. Similar to previous studies on in silico vaccine design [84–86], molecular docking analysis revealed strong binding affinity between Construct 1 and TLR-2, TLR-3, or TLR-4, with energy scores of −1237.4, −1337.5, and −1324.7, respectively. These findings suggest that Construct 1 has the potential to induce immune responses, particularly through TLR-3/TLR-4-mediated pathways. Molecular dynamic simulations were subsequently conducted to evaluate the stability and flexibility of the docked complex between Construct 1 and TLRs. Analysis of interaction energy, RMSD, RMSF, and PCA indicated high stability and low flexibility of Construct 1 upon binding to TLRs throughout the simulation period. Then 50S ribosomal protein L7/L12 of *Mycobacterium tuberculosis*, known for its strong induction of T-cell immune responses, was added as a TLR-4 agonist adjuvant [87]. It can activate immature dendritic cells and naïve T-cells via the 4 G8A receptor. This protein is typically added to the amino-terminal of vaccine constructs using suitable linkers [76, 88–95], thus it was also fused at the N-terminus of Construct 1. The adjuvant has been shown to induce dendritic cell maturation, TNF-alpha production, IL-1beta, and IL-6 pro-inflammatory cytokines, which activate T-cells to instigate cellular immune responses [87]. Here, it significantly improved the stability and immunogenicity of Construct 1.

SARS-CoV-2 is prone to mutations, which are evident in selected epitope-rich fragments of the virus. Mutations such as Q52H, A67V, ΔH69/V70, G75V, G339D, G339H, R346K, L452Q, L452R, Y453F, F456L, N460K, and S477N are associated with the fragments S1, S2, S3, and S4 (see Supplementary Table 5). All these mutations are manually curated in the UniProt database, except for ΔH69/V70 in Fragment S1 and L452R and Y453F in Fragment S4, which have published evidence. The ΔH69/V70 mutation may compensate for reduced infectivity in RBD escapes mutants and is observed in strains such as Alpha, Eta, 19B, and several Omicron variants (BA.1, BA.4, BA.5, BQ.1.1) [96]. Mutations L452R and Y453F, linked to immune evasion and increased infectivity, are found in strains including Delta, Epsilon, Kappa, Lambda, 19B, and multiple Omicron variants [97]. Other mutations in fragments M (positions 1–20), N2 (positions 151–174), and E (positions 1–17) are curated without published evidence of their impact on viral virulence (see Supplementary Table 5). These mutations may enable the virus to overcome herd immunity, infect unvaccinated individuals, or facilitate vaccine escape, raising the risk of severe disease or death. However, most studies indicate that current vaccines remain effective against circulating variants, protecting against severe to moderate disease outcomes [98]. For the proposed vaccine design, we used the reference sequences of key wild-type (WT) SARS-CoV-2 proteins, which correspond to the original Wuhan strain. By targeting multiple epitopes across these conserved proteins, our vaccine aims to maintain efficacy even as new mutations arise. Nonetheless, clinical trials are necessary to confirm its efficacy against current variants. Periodic updates or *de novo* designs may be required to keep pace with the ongoing evolution of SARS-CoV-2. *De novo* design would involve identifying new epitopes based on the mutated sequences of the novel strain, performing in silico analysis for epitope selection and population coverage, and incorporating these epitopes into a new vaccine construct. This approach ensures that the vaccine remains effective against newly circulating variants.

The immunoinformatics characteristics of the final vaccine were assessed using the C-ImmSim Server. Predicted results showed consistent population levels of active macrophages and dendritic cells (DCs), indicative of innate immune response activation upon antigen exposure. DCs, potent antigen-presenting cells (APCs), can stimulate immature T-cells to facilitate adaptive immunity. In silico immune response simulation predicted significant activation of helper T-cells postvaccination, leading to the release of IFN-*γ* and TNF-*α* cytokines. Moreover, the vaccine exhibited activation of B lymphocytes, cytotoxic T lymphocytes, and memory cell formation, suggesting a promising antiviral response in silico.

The finalized nucleotide sequence of the multiepitope vaccine was cloned into one of the taRNA system's vectors as TR-RNA, with another vector encoding nrRNA for the in-transacting VEEV replicase. This system enables continuous expression of the multiepitope antigen in the cytoplasm, enhancing immune responses involving APCs or antibodies [99]. Notable mRNA vaccines for COVID-19 include BNT162b1 [100], BNT162b2 [101], and mRNA-1273, which have shown efficacy in clinical trials [102]. While these vaccines primarily encode natural spike protein sequences in a nonreplicating approach, incorporating epitope prediction into RNA vaccine designs offers the potential for improving safety and immunogenicity. In the current study, several structural proteins of SARS-Cov-2 were used for epitope prediction for both B-cell and T-cell lymphocytes for enhancing humoral responses alongside T-cell immunogenicity and cross-reactivity. Computational tools offered the best combination of epitope sequences to put into an ORF and intracellularly amplify and express by the taRNA system. This system, although not systematically explored for infectious disease vaccines, offers advantages over the traditional RNA vaccine platforms and the saRNA system [103]. The split-vector system enhances safety, efficiency, and versatility by minimizing unknown replicative risks and allows independent optimization of antigen delivery and replicase activity. Additionally, the taRNA system promises easy, cost-efficient manufacturing by efficiently amplifying TR-RNA and boosting protein production for a potent immune response. Recent studies support the potential of taRNA-based vaccines as robust prophylactic options in preclinical models [104].

Overall, the vaccine's stability and immunogenicity have been validated, yet clinical trials are needed to confirm its efficacy. Consistency between immunoinformatics predictions and real-world experimental outcomes is emphasized in subsequent studies on multiepitope vaccines against SARS-CoV-2 [105–110]. Immunoinformatics has significantly advanced immunological research by offering cost-effective and precise methods, streamlining processes, and aiding targeted vaccine development. However, in vivo experimental evaluations are critical to ensure the vaccine's effectiveness and safety [111].

## Figures and Tables

**Table 1:** Predicted epitopes and overlapping fragments of structural proteins of SARS-CoV-2.

Protein	Overlapping epitope sequence (epitope-rich Fragment)	T-cell HLA-I (NetMHCPan/TepiTool)	T-cell HLA-II (TepiTool/consensus)	T Cell HLA-II (NetMHCIIpan)	B Cell consensus epitopes	Domain
Spike	TQDLFLPFFSNVTWFHAIHVSGTNG	—	LPFFSNVTWFHAIHV	LFLPFFSNVTWFHAI	VTWFHAIHVSGTNG	NTD
	TLDSKTQSLLIVNNATNVVIKVCE	TLDSKTQSL	SLLIVNNATNVVIKV	SLLIVNNATNVVIKV	—	NTD
	EFVFKNIDGYFKIYS	EFVFKNIDGYFKIYS	EFVFKNIDGYFKIYS	—	—	NTD
	CPFGEVFNATRFASVYAWNRKRISN	—	VFNATRFASVYAWNR	FGEVFNATRFASVYA	VFNATRFASVYAWNRKRISN	RBD
	CVADYSVLYNSASFSTFKC	CVADYSVLY	—	DYSVLYNSASFSTFK	—	RBD
	YLYRLFRKSNLKPFERDISTEIYQAGST	—	YLYRLFRKSNLKPFE	—	YLYRLFRKSNLKPFE	RBD
	PSKRSFIEDLLFNKVTLADAGFIK	LLFNKVTLA	LLFNKVTLADAGFIK	KRSFIEDLLFNKVTL	—	Cleavage site
	GAISSVLNDILSRLDKVEAEV	VLNDILSRL	GAISSVLNDILSRLD	—	—	HR1
	IWLGFIAGLIAIVMV	IWLGFIAGLIAIVMV	IWLGFIAGLIAIVMV	—	—	TM

Membrane	MADSNGTITVEELKKLLEQW	DSNGTITV	ADSNGTITVEELKKL	—	—	M

Nucleocapsid	INTNSSPDDQIGYYRRATRRIRGGDGKMKDLSPR	SSPDDQIGYY	DQIGYYRRATRRIRG	DQIGYYRRATRRIRG	INTNSSPDDQIG	NTD
	PANNAAIVLQLPQGTTLPKGFYAE	LQLPQGTTL	AAIVLQLPQGTTLPK	NAAIVLQLPQGTTLP	GTTLPKG	NTD

Envelope	MYSFVSEETGTLIVNSV	TLIVNSVLL	SEETGTLIVNSVLLF	—	—	E protein

**Table 2:** The immunogenicity and physicochemical properties of consensus epitope-rich fragments.

Consensus fragment	Immunogenicity	Aliphatic index	GRAVY	Isoelectric point (pI)	Molecular weight	Instability index
Fragment S1	0.72742	74.00	0.244	5.90	2836.16	9.80
Fragment S2	0.43324	101.67	0.087	8.90	2710.17	31.55
Fragment S3	0.62368	50.80	−0.348	10.05	2934.33	7.27
Fragment S4	0.03525	73.21	−0.739	9.40	3396.85	18.51
Fragment M	0.12739	97.50	−0.540	4.00	2332.61	26.74
Fragment N2	0.01933	93.75	0.017	6.42	2513.88	35.96
Fragment E	0.04893	97.06	0.565	3.79	1876.11	38.86

**Table 3:** Docking results of each epitope-rich fragment (ligand) with HLA alleles (receptor) by using AutoDock4 software.

Ligand name	HLA allele (receptor Protein)	Intramolecular energy (kJ/mol)	Intermolecular energy (kJ/mol)
Fragment S1	HLA-A^∗^02:01	−19.09	−11.99
	HLA-DRB1^∗^01:01, DRA^∗^01:01	−2166	−7.53
Fragment S2	HLA-A^∗^02:01	−16.56	−10.7
	HLA-DRB1^∗^01:01, DRA ^∗^01:01	−18.46	−7.82
Fragment S3	HLA-A ^∗^02:01	−13.59	−13.31
	HLA-DRB1^∗^01:01, DRA ^∗^01:01	−18.41	−6.03
Fragment S4	HLA-A^∗^02:01	−19.18	−9.84
	HLA-DRB1^∗^01:01, DRA^∗^01:01	−17.24	−7.13
Fragment E	HLA-A ^∗^02:01	−11.74	−9.43
	HLA-DRB1 ^∗^01:01, DRA ^∗^01:01	−12.47	−6.53
Fragment M	HLA-A ^∗^02:01	−10.71	−8.57
	HLA-DRB1 ^∗^01:01, DRA ^∗^01:01	−13.15	−8.04
Fragment N2	HLA-A ^∗^02:01	−16.72	−9.79
	HLA-DRB1 ^∗^01:01, DRA ^∗^01:01	−18.01	−9.45

**Table 4:** Physicochemical properties of Construct 1 compared to the final vaccine candidate.

Features	Assessment for Construct 1	Assessment for adjuvant-Construct 1
Antigenicity	Probable ANTIGEN (0.3091 in VaxiJenv2.0 and 0.6673 in ANTIGENPro)	Probable ANTIGEN (0.3472 in VaxiJenv2.0 and 0.7228 in ANTIGENPro)

Allergenicity	Probable nonallergen (AllerTOPv2.0)	Probable nonallergen (AllerTOPv2.0)

Solubility (protein-sol)	0.437 (soluble)	0.660 (soluble)

Number of amino acids	172	307

Molecular weight	18925.35 Dalton	32818.34 Dalton

Isoelectric point (pI)	6.94	6.02

Total number of atoms	2644	4643

Formula	C_859_H_1304_N_226_O_252_S_3_	C_1480_H_2327_N_381_O_450_S_5_

Estimated half-life	2.8 hours (mammalian reticulocytes, in vitro)	30 hours (mammalian reticulocytes, in vitro)
	10 min (yeast, in vivo)	>20 hours (yeast, in vivo)
	2 min (*Escherichia coli*, in vivo)	>10 hours (*Escherichia coli*, in vivo)

**Table 5:** Population coverage calculation result.

Population/area	Class combined			Class I			Class II		
	Coverage^a^ (%)	average_hit^b^	pc90^c^	Coverage^a^ (%)	average_hit^b^	pc90^c^	Coverage^a^ (%)	average_hit^b^	pc90^c^
World average	94.46	10.16	2.25	69.53	1.88	0.33	81.81	8.28	1.1

^a^projected population coverage, ^b^average number of epitope hits/HLA combinations recognized by the population, ^c^minimum number of epitope hits/HLA combinations recognized by 90% of the population.

## Data Availability

The data used to support the findings of this study are included in the article.

## Supplementary Materials

Supplementary Materials Supplementary Tables 1, 2, 3, and 4: Predicted discontinuous B-cell epitopes of the Spike, Membrane, Nucleocapsid, and Envelope proteins, respectively, using ElliPro-IEDB analysis. Supplementary Table 5: Variants associated with the selected epitope-rich fragments. Supplementary Figure 1: Population coverage of the selected epitopes.

## References

[1] Satarker S., Nampoothiri M. (2020). Structural proteins in severe acute respiratory syndrome coronavirus-2. *Archives of Medical Research*.

[2] Fiolet T., Kherabi Y., MacDonald C.-J., Ghosn J., Peiffer-Smadja N. (2022). Comparing COVID-19 vaccines for their characteristics, efficacy, and effectiveness against SARS-CoV-2 and variants of concern: a narrative review. *Clinical Microbiology and Infection*.

[3] Nafian F., Nafian S., Soleymani G. (2021). Next-generation vaccines based on self-amplifying RNA.

[4] Ballesteros-Briones M. C., Silva-Pilipich N., Herrador-Cañete G., Vanrell L., Smerdou C. (2020). A new generation of vaccines based on alphavirus self-amplifying RNA. *Current opinion in virology*.

[5] Thomas S. (2020). The structure of the membrane protein of SARS-CoV-2 resembles the sugar transporter SemiSWEET. *Pathogens and Immunity*.

[6] Herrera N. G., Morano N. C., Celikgil A. (2020). Characterization of the SARS-CoV-2 S protein: biophysical, biochemical, structural, and antigenic analysis. *ACS Omega*.

[7] Hossain M. G., Tang Yd, Akter S., Zheng C. (2022). Roles of the polybasic furin cleavage site of spike protein in SARS‐CoV‐2 replication, pathogenesis, and host immune responses and vaccination. *Journal of Medical Virology*.

[8] Xia X. (2021). Domains and functions of spike protein in Sars-Cov-2 in the context of vaccine design. *Viruses*.

[9] Kumavath R., Barh D., Andrade B. S. (2021). The spike of SARS-CoV-2: uniqueness and applications. *Frontiers in Immunology*.

[10] Malik Y. A. (2020). Properties of coronavirus and SARS-CoV-2. *Malaysian Journal of Pathology*.

[11] Ruch T. R., Machamer C. E. (2012). The coronavirus E protein: assembly and beyond. *Viruses*.

[12] Mukherjee S., Bhattacharyya D., Bhunia A. (2020). Host-membrane interacting interface of the SARS coronavirus envelope protein: immense functional potential of C-terminal domain. *Biophysical Chemistry*.

[13] Patra R., Chandra Das N., Mukherjee S. (2021). Targeting human TLRs to combat COVID‐19: a solution?. *Journal of Medical Virology*.

[14] Choudhury A., Mukherjee S. (2020). In silico studies on the comparative characterization of the interactions of SARS‐CoV‐2 spike glycoprotein with ACE‐2 receptor homologs and human TLRs. *Journal of Medical Virology*.

[15] Choudhury A., Das N. C., Patra R., Mukherjee S. (2021). In silico analyses on the comparative sensing of SARS‐CoV‐2 mRNA by the intracellular TLRs of humans. *Journal of Medical Virology*.

[16] Lei X., Dong X., Ma R. (2020). Activation and evasion of type I interferon responses by SARS-CoV-2. *Nature Communications*.

[17] Mukherjee S. (2022). Toll-like receptor 4 in COVID-19: friend or foe?. *Future Virology*.

[18] Choudhury A., Mukherjee G., Mukherjee S. (2021). Chemotherapy vs. Immunotherapy in combating nCOVID19: an update. *Human Immunology*.

[19] Zhang Y., Zeng G., Pan H. (2021). Safety, tolerability, and immunogenicity of an inactivated SARS-CoV-2 vaccine in healthy adults aged 18–59 years: a randomised, double-blind, placebo-controlled, phase 1/2 clinical trial. *The Lancet Infectious Diseases*.

[20] Wu Z., Hu Y., Xu M. (2021). Safety, tolerability, and immunogenicity of an inactivated SARS-CoV-2 vaccine (CoronaVac) in healthy adults aged 60 years and older: a randomised, double-blind, placebo-controlled, phase 1/2 clinical trial. *The Lancet Infectious Diseases*.

[21] Keech C., Albert G., Cho I. (2020). Phase 1–2 trial of a SARS-CoV-2 recombinant spike protein nanoparticle vaccine. *New England Journal of Medicine*.

[22] Voysey M., Clemens S. A. C., Madhi S. A. (2021). Safety and efficacy of the ChAdOx1 nCoV-19 vaccine (AZD1222) against SARS-CoV-2: an interim analysis of four randomised controlled trials in Brazil, South Africa, and the UK. *The Lancet*.

[23] Self W. H., Tenforde M. W., Rhoads J. P. (2021). Comparative effectiveness of Moderna, Pfizer-BioNTech, and Janssen (Johnson and Johnson) vaccines in preventing COVID-19 hospitalizations among adults without immunocompromising conditions—United States, March-August 2021. *MMWR. Morbidity and Mortality Weekly Report*.

[24] Logunov D. Y., Dolzhikova I. V., Zubkova O. V. (2020). Safety and immunogenicity of an rAd26 and rAd5 vector-based heterologous prime-boost COVID-19 vaccine in two formulations: two open, non-randomised phase 1/2 studies from Russia. *The Lancet*.

[25] Chakraborty C., Agoramoorthy G. (2020). India’s cost-effective COVID-19 vaccine development initiatives. *Vaccine*.

[26] Polack F. P., Thomas S. J., Kitchin N. (2020). Safety and efficacy of the BNT162b2 mRNA Covid-19 vaccine. *New England Journal of Medicine*.

[27] Jackson L. A., Anderson E. J., Rouphael N. G. (2020). An mRNA vaccine against SARS-CoV-2—preliminary report. *New England Journal of Medicine*.

[28] Bateman A., Martin M. J., Orchard S. (2021). UniProt: the universal protein knowledgebase in 2021. *Nucleic Acids Research*.

[29] Kar T., Narsaria U., Basak S. (2020). A candidate multi-epitope vaccine against SARS-CoV-2. *Scientific Reports*.

[30] Paul S., Lindestam Arlehamn C. S., Scriba T. J. (2015). Development and validation of a broad scheme for prediction of HLA class II-restricted T cell epitopes. *Journal of Immunological Methods*.

[31] Paul S., Sidney J., Sette A., Peters B. (2016). TepiTool: a pipeline for computational prediction of T cell epitope candidates. *Current Protocols in Immunology*.

[32] Vita R., Mahajan S., Overton J. A. (2019). The immune epitope database (IEDB): 2018 update. *Nucleic Acids Research*.

[33] Lundegaard C., Lund O., Nielsen M. (2008). Accurate approximation method for prediction of class I MHC affinities for peptides of length 8, 10 and 11 using prediction tools trained on 9mers. *Bioinformatics*.

[34] Reynisson B., Alvarez B., Paul S., Peters B., Nielsen M. (2020). NetMHCpan-4.1 and NetMHCIIpan-4.0: improved predictions of MHC antigen presentation by concurrent motif deconvolution and integration of MS MHC eluted ligand data. *Nucleic Acids Research*.

[35] Yalcinkaya B. (2021). Immunoinformatic design and evaluation of a novel multi-epitope peptide vaccine targeting SARS-CoV-2 structural and nonstructural antigens. https://github.com/berkyalcinkaya/Computational-SARS-CoV-2-Vaccine-Design.

[36] Larsen J. E., Lund O., Nielsen M. (2006). Improved method for predicting linear B-cell epitopes. *Immunome Research*.

[37] Ponomarenko J. V., Van Regenmortel M. H. (2009). B cell epitope prediction. *Structural bioinformatics*.

[38] Chen J., Liu H., Yang J., Chou K.-C. (2007). Prediction of linear B-cell epitopes using amino acid pair antigenicity scale. *Amino Acids*.

[39] Fleri W., Paul S., Dhanda S. K. (2017). The immune epitope database and analysis resource in epitope discovery and synthetic vaccine design. *Frontiers in Immunology*.

[40] Stecher G., Tamura K., Kumar S. (2020). Molecular evolutionary genetics analysis (MEGA) for macOS. *Molecular Biology and Evolution*.

[41] Calis J. J., Maybeno M., Greenbaum J. A. (2013). Properties of MHC class I presented peptides that enhance immunogenicity. *PLoS Computational Biology*.

[42] Walker J. M. (2005). *The Proteomics Protocols Handbook*.

[43] Lamiable A., Thévenet P., Rey J., Vavrusa M., Derreumaux P., Tufféry P. (2016). PEP-FOLD3: faster de novo structure prediction for linear peptides in solution and in complex. *Nucleic Acids Research*.

[44] Burley S. K., Bhikadiya C., Bi C. (2021). RCSB Protein Data Bank: powerful new tools for exploring 3D structures of biological macromolecules for basic and applied research and education in fundamental biology, biomedicine, biotechnology, bioengineering, and energy sciences. *Nucleic Acids Research*.

[45] Bitencourt-Ferreira G., Pintro V. O., de Azevedo W. F. (2019). Docking with autodock4. *Methods in Molecular Biology*.

[46] Yang J., Yan R., Roy A., Xu D., Poisson J., Zhang Y. (2015). The I-TASSER Suite: protein structure and function prediction. *Nature Methods*.

[47] Robertson M. J., Tirado-Rives J., Jorgensen W. L. (2015). Improved peptide and protein torsional energetics with the OPLS-AA force field. *Journal of Chemical Theory and Computation*.

[48] Desta I. T., Porter K. A., Xia B., Kozakov D., Vajda S. (2020). Performance and its limits in rigid-body protein-protein docking. *Structure*.

[49] Schrodinger L. L. C. (2015). *The PyMOL Molecular Graphics System*.

[50] Feig M. (2016). Local protein structure refinement via molecular dynamics simulations with locPREFMD. *Journal of Chemical Information and Modeling*.

[51] Doytchinova I. A., Flower D. R. (2007). VaxiJen: a server for prediction of protective antigens, tumor antigens, and subunit vaccines. *BMC Bioinformatics*.

[52] Dimitrov I., Bangov I., Flower D. R., Doytchinova I. (2014). AllerTOP v. 2—a server for in silico prediction of allergens. *Journal of Molecular Modeling*.

[53] Bui H.-H., Sidney J., Dinh K., Southwood S., Newman M. J., Sette A. (2006). Predicting population coverage of T-cell epitope-based diagnostics and vaccines. *BMC Bioinformatics*.

[54] Castiglione F., Deb D., Srivastava A. P., Liò P., Liso A. (2021). From infection to immunity: understanding the response to SARS-CoV2 through in-silico modeling. *Frontiers in Immunology*.

[55] Castiglione F., Mantile F., De Berardinis P., Prisco A. (2012). How the interval between prime and boost injection affects the immune response in a computational model of the immune system. *Computational and Mathematical Methods in Medicine*.

[56] Paul S., Weiskopf D., Angelo M. A., Sidney J., Peters B., Sette A. (2013). HLA class I alleles are associated with peptide-binding repertoires of different size, affinity, and immunogenicity. *The Journal of Immunology*.

[57] Ponomarenko J., Bui H.-H., Li W. (2008). ElliPro: a new structure-based tool for the prediction of antibody epitopes. *BMC Bioinformatics*.

[58] Zheng W., Zhang C., Li Y., Pearce R., Bell E. W., Zhang Y. (2021). Folding non-homologous proteins by coupling deep-learning contact maps with I-TASSER assembly simulations. *Cell reports methods*.

[59] Mahmoodi S., Amirzakaria J. Z., Ghasemian A. (2023). In silico design and validation of a novel multi-epitope vaccine candidate against structural proteins of Chikungunya virus using comprehensive immunoinformatics analyses. *PLoS One*.

[60] Orlandini von Niessen A. G., Poleganov M. A., Rechner C. (2019). Improving mRNA-based therapeutic gene delivery by expression-augmenting 3′ UTRs identified by cellular library screening. *Molecular Therapy*.

[61] Sahin U., Muik A., Derhovanessian E. (2020). COVID-19 vaccine BNT162b1 elicits human antibody and TH1 T cell responses. *Nature*.

[62] Mo C., Li X., Wu Q. (2023). SARS-CoV-2 mRNA vaccine requires signal peptide to induce antibody responses. *Vaccine*.

[63] Lin G., Zhang S., Zhong Y. (2021). Community evidence of severe acute respiratory syndrome coronavirus 2 (SARS-CoV-2) transmission through air. *Atmospheric Environment*.

[64] Samad A., Ahammad F., Nain Z. (2022). Designing a multi-epitope vaccine against SARS-CoV-2: an immunoinformatics approach. *Journal of Biomolecular Structure and Dynamics*.

[65] Lim H. X., Lim J., Jazayeri S. D., Poppema S., Poh C. L. (2021). Development of multi-epitope peptide-based vaccines against SARS-CoV-2. *Biomedical Journal*.

[66] Jyotisha S. S., Singh S., Qureshi I. A. (2022). Multi-epitope vaccine against SARS-CoV-2 applying immunoinformatics and molecular dynamics simulation approaches. *Journal of Biomolecular Structure and Dynamics*.

[67] Safavi A., Kefayat A., Mahdevar E., Abiri A., Ghahremani F. (2020). Exploring the out-of-sight antigens of SARS-CoV-2 to design a candidate multi-epitope vaccine by utilizing immunoinformatics approaches. *Vaccine*.

[68] Yu M., Zhu Y., Li Y. (2022). Design of a recombinant multivalent epitope vaccine based on SARS-CoV-2 and its variants in immunoinformatics approaches. *Frontiers in Immunology*.

[69] Lokman S. M., Rasheduzzaman M., Salauddin A. (2020). Exploring the genomic and proteomic variations of SARS-CoV-2 spike glycoprotein: a computational biology approach. *Infection, Genetics and Evolution*.

[70] Zecha J., Lee C.-Y., Bayer F. P. (2020). Data, reagents, assays and merits of proteomics for SARS-CoV-2 research and testing. *Molecular and Cellular Proteomics*.

[71] Li J., Guo M., Tian X. (2021). Virus-host interactome and proteomic survey reveal potential virulence factors influencing SARS-CoV-2 pathogenesis. *Médica Sur*.

[72] Yadav R., Chaudhary J. K., Jain N. (2021). Role of structural and non-structural proteins and therapeutic targets of SARS-CoV-2 for COVID-19. *Cells*.

[73] Chen H. Z., Tang L. L., Yu X. L., Zhou J., Chang Y. F., Wu X. (2020). Bioinformatics analysis of epitope-based vaccine design against the novel SARS-CoV-2. *Infectious Diseases of Poverty*.

[74] Samrat S. K., Tharappel A. M., Li Z., Li H. (2020). Prospect of SARS-CoV-2 spike protein: potential role in vaccine and therapeutic development. *Virus Research*.

[75] Baruah V., Bose S. (2020). Immunoinformatics‐aided identification of T cell and B cell epitopes in the surface glycoprotein of 2019‐nCoV. *Journal of Medical Virology*.

[76] Abraham Peele K., Srihansa T., Krupanidhi S., Ayyagari V. S., Venkateswarulu T. (2021). Design of multi-epitope vaccine candidate against SARS-CoV-2: a in-silico study. *Journal of Biomolecular Structure and Dynamics*.

[77] Sanami S., Zandi M., Pourhossein B. (2020). Design of a multi-epitope vaccine against SARS-CoV-2 using immunoinformatics approach. *International Journal of Biological Macromolecules*.

[78] Khan A., Khan T., Ali S. (2021). SARS-CoV-2 new variants: characteristic features and impact on the efficacy of different vaccines. *Biomedicine and Pharmacotherapy*.

[79] Wang C., Zhou X., Wang M., Chen X. (2021). The impact of SARS-CoV-2 on the human immune system and microbiome. *Infectious Microbes and Diseases*.

[80] Chai J., Cai Y., Pang C. (2021). Structural basis for SARS-CoV-2 envelope protein recognition of human cell junction protein PALS1. *Nature Communications*.

[81] Singh A., Thakur M., Sharma L. K., Chandra K. (2020). Designing a multi-epitope peptide-based vaccine against SARS-CoV-2. *Scientific Reports*.

[82] Sarkar B., Ullah M. A., Johora F. T., Taniya M. A., Araf Y. (2020). Immunoinformatics-guided designing of epitope-based subunit vaccines against the SARS Coronavirus-2 (SARS-CoV-2). *Immunobiology*.

[83] El-Zayat S. R., Sibaii H., Mannaa F. A. (2019). Toll-like receptors activation, signaling, and targeting: an overview. *Bulletin of the National Research Centre*.

[84] Das N. C., Patra R., Gupta P. S. S. (2021). Designing of a novel multi-epitope peptide-based vaccine against *Brugia malayi*: an in silico approach. *Infection, Genetics and Evolution*.

[85] Gorai S., Das N. C., Gupta P. S. S., Panda S. K., Rana M. K., Mukherjee S. (2022). Designing efficient multi-epitope peptide-based vaccine by targeting the antioxidant thioredoxin of bancroftian filarial parasite. *Infection, Genetics and Evolution*.

[86] Das N. C., Gupta P. S. S., Panda S. K., Rana M. K., Mukherjee S. (2023). Reverse vaccinology assisted design of a novel multi-epitope vaccine to target Wuchereria bancrofti cystatin: an immunoinformatics approach. *International Immunopharmacology*.

[87] Lee S. J., Shin S. J., Lee M. H. (2014). A potential protein adjuvant derived from *Mycobacterium tuberculosis* Rv0652 enhances dendritic cells-based tumor immunotherapy. *PLoS One*.

[88] Mishra K. K., Mishra A. K., Anand V., Pandey A., Budhwar S., Sharma D. C. (2023). Design of a multi-epitope vaccine against covid-19: an in silico approach. *Current Biotechnology*.

[89] Tourani M., Samavarchi Tehrani S., Movahedpour A. (2023). Design and evaluation of a multi-epitope vaccine for COVID-19: an in silico approach. *Health Science Monitor*.

[90] Devi A., Chaitanya N. S. (2021). In silico, designing of multi-epitope vaccine construct against human coronavirus infections. *Journal of Biomolecular Structure and Dynamics*.

[91] Alizadeh M., Amini-Khoei H., Tahmasebian S. (2022). Designing a novel multi-epitope vaccine against Ebola virus using reverse vaccinology approach. *Scientific Reports*.

[92] Ojha R., Singh S., Gupta N., Kumar K., Padhi A. K., Prajapati V. K. (2023). Multi-pathogen-based chimeric vaccine to fight against COVID-19 and concomitant coinfections. *Biotechnology Letters*.

[93] Martin W. R., Cheng F. (2022). A rational design of a multi-epitope vaccine against SARS-CoV-2 which accounts for the glycan shield of the spike glycoprotein. *Journal of Biomolecular Structure and Dynamics*.

[94] Kumar A., Rathi E., Kini S. G. (2022). Computational design of a broad-spectrum multi-epitope vaccine candidate against seven strains of human coronaviruses. *3 Biotech*.

[95] Maleki A., Russo G., Parasiliti Palumbo G. A., Pappalardo F. (2021). In silico design of recombinant multi-epitope vaccine against influenza A virus. *BMC Bioinformatics*.

[96] Meng B., Kemp S. A., Papa G. (2021). Recurrent emergence of SARS-CoV-2 spike deletion H69/V70 and its role in the Alpha variant B. 1.1. 7. *Cell Reports*.

[97] Motozono C., Toyoda M., Zahradnik J. (2021). SARS-CoV-2 spike L452R variant evades cellular immunity and increases infectivity. *Cell Host & Microbe*.

[98] Mistry P., Barmania F., Mellet J. (2021). SARS-CoV-2 variants, vaccines, and host immunity. *Frontiers in Immunology*.

[99] Cai X., Li J. J., Liu T., Brian O., Li J. (2021). Infectious disease mRNA vaccines and a review on epitope prediction for vaccine design. *Briefings in Functional Genomics*.

[100] Mulligan M. J., Lyke K. E., Kitchin N. (2020). Phase I/II study of COVID-19 RNA vaccine BNT162b1 in adults. *Nature*.

[101] Walsh E. E., Frenck R. W., Falsey A. R. (2020). Safety and immunogenicity of two RNA-based Covid-19 vaccine candidates. *New England Journal of Medicine*.

[102] Corbett K. S., Flynn B., Foulds K. E. (2020). Evaluation of the mRNA-1273 vaccine against SARS-CoV-2 in nonhuman primates. *New England Journal of Medicine*.

[103] Schmidt C., Haefner E., Gerbeth J. (2022). A taRNA vaccine candidate induces a specific immune response that protects mice against Chikungunya virus infections. *Molecular Therapy - Nucleic Acids*.

[104] Beissert T., Perkovic M., Vogel A. (2020). A trans-amplifying RNA vaccine strategy for induction of potent protective immunity. *Molecular Therapy*.

[105] Dong Y., Dai T., Wei Y., Zhang L., Zheng M., Zhou F. (2020). A systematic review of SARS-CoV-2 vaccine candidates. *Signal Transduction and Targeted Therapy*.

[106] Poh C. M., Carissimo G., Wang B. (2020). Two linear epitopes on the SARS-CoV-2 spike protein that elicit neutralising antibodies in COVID-19 patients. *Nature Communications*.

[107] Smith C. C., Olsen K. S., Gentry K. M. (2021). Landscape and selection of vaccine epitopes in SARS-CoV-2. *Genome Medicine*.

[108] Li Y., Ma M.-l, Lei Q. (2021). Linear epitope landscape of the SARS-CoV-2 Spike protein constructed from 1,051 COVID-19 patients. *Cell Reports*.

[109] Guirakhoo F., Kuo L., Peng J. (2020). A Novel SARS-CoV-2 multitope protein/peptide vaccine candidate is highly immunogenic and prevents lung infection in an AAV hACE2 mouse model and non-human primates. https://www.biorxiv.org/content/10.1101/2020.11.30.399154v2.

[110] Yarmarkovich M., Warrington J. M., Farrel A., Maris J. M. (2020). Identification of SARS-CoV-2 vaccine epitopes predicted to induce long-term population-scale immunity. *Cell Reports Medicine.*.

[111] Nafian F., Nafian S., Kamali Doust Azad B. (2020). Regulatory and biosafety challenges for vaccines. *Iranian Journal of Medical Microbiology*.

