# Supplementary material for: In Silico Design of a Trans-Amplifying RNA-Based Vaccine against SARS-CoV-2 Structural Proteins
Source: Adv Virol. 2024 Sep 30;2024:3418062. doi: 10.1155/2024/3418062 (PMC11459942; doi:10.1155/2024/3418062)
Supplement: Supplementary Materials — Supplementary Tables 1, 2, 3, and 4: Predicted discontinuous B-cell epitopes of the Spike, Membrane, Nucleocapsid, and Envelope proteins, respectively, using ElliPro-IEDB analysis. Supplementary Table 5: Variants associated with the selected epitope-rich fragments. Supplementary Figure 1: Population coverage of the selected epitopes. [file 3418062.f1.zip › Supplementary Table 1.docx]

Supplementary Table 1. Predicted discontinuous B-cell epitopes of the Spike protein using ElliPro-IEDB analysis on PDB ID 6X29 (Positions 16-1208.

| **No.** | **Residues** | **Number of residues** | **Score** |
| --- | --- | --- | --- |
| 1 | A:Y707, A:S708, A:N709, A:N710, A:S711, A:I712, A:A713, A:I714, A:P715, A:T716, A:N717, A:A783, A:Q784, A:V785, A:K786, A:Q787, A:I788, A:Y789, A:K790, A:T791, A:P792, A:P793, A:I794, A:K795, A:D796, A:F797, A:G798, A:G799, A:F800, A:N801, A:F802, A:S803, A:Q804, A:I805, A:L806, A:P807, A:D808, A:P809, A:S810, A:K811, A:P812, A:S813, A:K814, A:R815, A:P863, A:L864, A:L865, A:E868, A:M869, A:Q872, A:Y873, A:S875, A:A876, A:A879, A:G880, A:I882, A:T883, A:S884, A:G885, A:W886, A:T887, A:F888, A:G889, A:A890, A:G891, A:A892, A:A893, A:L894, A:Q895, A:I896, A:P897, A:F898, A:A899, A:M900, A:Q901, A:M902, A:A903, A:Y904, A:F906, A:N907, A:G908, A:I909, A:G910, A:V911, A:T912, A:Q913, A:N914, A:V915, A:L916, A:Y917, A:E918, A:N919, A:Q920, A:K921, A:L922, A:I923, A:A924, A:N925, A:L1034, A:G1035, A:Q1036, A:Q1071, A:E1072, A:K1073, A:N1074, A:F1075, A:T1076, A:T1077, A:A1078, A:P1079, A:A1080, A:I1081, A:C1082, A:H1083, A:D1084, A:G1085, A:K1086, A:A1087, A:H1088, A:F1089, A:P1090, A:R1091, A:G1093, A:V1094, A:F1095, A:V1096, A:S1097, A:N1098, A:G1099, A:T1100, A:H1101, A:W1102, A:F1103, A:V1104, A:T1105, A:Q1106, A:R1107, A:N1108, A:F1109, A:Y1110, A:E1111, A:P1112, A:Q1113, A:I1114, A:I1115, A:T1116, A:T1117, A:D1118, A:N1119, A:T1120, A:F1121, A:V1122, A:S1123, A:G1124, A:N1125, A:C1126, A:D1127, A:V1128, A:V1129, A:I1130, A:G1131, A:I1132, A:V1133, A:N1134, A:N1135, A:T1136, A:V1137, A:Y1138, A:D1139, A:P1140, A:L1141, A:Q1142, A:P1143, A:E1144, A:L1145, A:D1146, A:S1147 | 177 | 0.735 |
| 2 | A:I326, A:R328, A:F329, A:P330, A:N331, A:I332, A:T333, A:N334, A:L335, A:C336, A:P337, A:F338, A:G339, A:E340, A:V341, A:F342, A:N343, A:A344, A:T345, A:R346, A:F347, A:A348, A:S349, A:V350, A:Y351, A:A352, A:W353, A:N354, A:R355, A:K356, A:R357, A:I358, A:S359, A:N360, A:C361, A:V362, A:A363, A:D364, A:V367, A:L368, A:S371, A:A372, A:S373, A:F374, A:S375, A:T376, A:Y380, A:C391, A:F392, A:T393, A:N394, A:V395, A:Y396, A:A397, A:D398, A:S399, A:F400, A:V401, A:I402, A:R403, A:G404, A:D405, A:E406, A:V407, A:R408, A:Q409, A:I410, A:A411, A:P412, A:G413, A:Q414, A:T415, A:G416, A:K417, A:I418, A:A419, A:D420, A:Y421, A:N422, A:Y423, A:K424, A:L425, A:P426, A:D427, A:D428, A:F429, A:T430, A:V433, A:I434, A:A435, A:W436, A:N437, A:S438, A:N439, A:N440, A:L441, A:D442, A:S443, A:K444, A:G447, A:N448, A:Y449, A:N450, A:Y451, A:L452, A:Y453, A:R454, A:K462, A:P463, A:F464, A:E465, A:R466, A:D467, A:I468, A:Y489, A:F490, A:P491, A:L492, A:Q493, A:S494, A:Y495, A:G496, A:F497, A:Q498, A:P499, A:T500, A:N501, A:V503, A:G504, A:Y505, A:Q506, A:P507, A:Y508, A:R509, A:V510, A:V511, A:V512, A:L513, A:S514, A:E516, A:L517, A:L518, A:H519, A:A520, A:P521, A:A522, A:T523, A:V524, A:C525, A:G526, A:P527, A:K528, A:K529, A:S530, A:T531, A:N532, A:L533, A:V534, A:K535, A:N536, A:K537, A:N544, A:T553, A:E554, A:S555, A:N556, A:K557, A:F559, A:L560, A:P561, A:F562, A:Q563, A:Q564, A:V576, A:R577, A:D578, A:P579, A:Q580, A:T581, A:L582, A:E583, A:I584, A:L585 | 183 | 0.735 |
| 3 | A:A27, A:Y28, A:T29, A:N30, A:S31, A:F32, A:R34, A:F59, A:S60, A:V62, A:T63, A:W64, A:F65, A:H66, A:A67, A:I68, A:H69, A:D80, A:N81, A:P82, A:V83, A:L84, A:P85, A:N87, A:F92, A:A93, A:S94, A:T95, A:E96, A:K97, A:S98, A:N99, A:I100, A:I101, A:R102, A:G103, A:W104, A:I105, A:F106, A:G107, A:T108, A:T109, A:L110, A:D111, A:S112, A:K113, A:T114, A:Q115, A:S116, A:L117, A:L118, A:I119, A:V120, A:N121, A:N122, A:A123, A:T124, A:N125, A:V126, A:V127, A:I128, A:K129, A:V130, A:C131, A:E132, A:F133, A:Q134, A:F135, A:C136, A:N137, A:D138, A:P139, A:F140, A:L141, A:G142, A:V143, A:N165, A:C166, A:T167, A:F168, A:E169, A:Y170, A:V171, A:S172, A:F186, A:K187, A:N188, A:L189, A:R190, A:E191, A:F192, A:S205, A:K206, A:H207, A:T208, A:P209, A:I210, A:N211, A:L212, A:V213, A:R214, A:D215, A:L216, A:P217, A:Q218, A:G219, A:S221, A:A222, A:L223, A:I233, A:N234, A:I235, A:T236, A:R237, A:F238, A:Q239, A:T240, A:L241, A:L242, A:A243, A:L244, A:H245, A:A263, A:A264, A:Y265, A:Y266, A:V267, A:G268, A:Y269 | 129 | 0.725 |
| 4 | A:N703, A:S704, A:V705, A:A706 | 4 | 0.632 |
| 5 | A:G744, A:D745, A:S746, A:T747, A:E748, A:S750, A:N751, A:L754, A:Q755, A:G757, A:S758 | 11 | 0.539 |
| 6 | A:L226, A:V227, A:L229, A:P230, A:I231 | 5 | 0.523 |
