# Supplementary material for: In Silico Design of a Trans-Amplifying RNA-Based Vaccine against SARS-CoV-2 Structural Proteins
Source: Adv Virol. 2024 Sep 30;2024:3418062. doi: 10.1155/2024/3418062 (PMC11459942; doi:10.1155/2024/3418062)
Supplement: Supplementary Materials — Supplementary Tables 1, 2, 3, and 4: Predicted discontinuous B-cell epitopes of the Spike, Membrane, Nucleocapsid, and Envelope proteins, respectively, using ElliPro-IEDB analysis. Supplementary Table 5: Variants associated with the selected epitope-rich fragments. Supplementary Figure 1: Population coverage of the selected epitopes. [file 3418062.f1.zip › Supplementary Table 2.docx]

Supplementary Table 2. Predicted discontinuous B-cell epitopes of the Membrane protein using ElliPro-IEDB analysis on PDB ID 7VGR (Positions 1-222).

| **No.** | **Residues** | **Number of residues** | **Score** |
| --- | --- | --- | --- |
| 1 | A:T9, A:V10, A:E11, A:E12, A:L13, A:K14, A:K15, A:L16, A:L17, A:E18, A:Q19, A:W20 | 12 | 0.939 |
| 2 | A:A63, A:V66, A:L67, A:A68, A:A69, A:V70, A:Y71, A:R72, A:I73, A:N74, A:W75, A:I76, A:T77, A:G78, A:G79, A:I80, A:A81, A:I82, A:A83, A:M84, A:A85, A:C86 | 22 | 0.771 |
| 3 | A:N21, A:L22, A:V23, A:I24, A:G25, A:F26, A:F28, A:L29, A:I32, A:C33, A:Q36, A:F37, A:Y39, A:N41, A:R42 | 15 | 0.657 |
| 4 | A:A142, A:V143, A:I144, A:L145, A:R146, A:G147, A:H148, A:R150, A:G153, A:G157, A:R158, A:C159, A:D160, A:I161, A:K162, A:D163, A:L164, A:P165, A:K166, A:A171, A:T172, A:S173, A:R174, A:T175, A:L176, A:Y178, A:K180, A:L181, A:G182, A:A183, A:S184, A:Q185, A:R186, A:V187, A:A188, A:G189, A:D190, A:S191, A:G192, A:F193, A:A194, A:Y199, A:R200, A:I201, A:G202, A:N203, A:Y204 | 47 | 0.629 |
| 5 | A:A104, A:K205, A:L206 | 3 | 0.618 |
| 6 | A:E135, A:S136, A:E137, A:L138, A:V139, A:R198 | 6 | 0.517 |
