# Supplementary material for: In Silico Design of a Trans-Amplifying RNA-Based Vaccine against SARS-CoV-2 Structural Proteins
Source: Adv Virol. 2024 Sep 30;2024:3418062. doi: 10.1155/2024/3418062 (PMC11459942; doi:10.1155/2024/3418062)
Supplement: Supplementary Materials — Supplementary Tables 1, 2, 3, and 4: Predicted discontinuous B-cell epitopes of the Spike, Membrane, Nucleocapsid, and Envelope proteins, respectively, using ElliPro-IEDB analysis. Supplementary Table 5: Variants associated with the selected epitope-rich fragments. Supplementary Figure 1: Population coverage of the selected epitopes. [file 3418062.f1.zip › Supplementary Table 3.docx]

Supplementary Table 3. Predicted discontinuous B-cell epitopes of the Nucleocapsid protein using ElliPro-IEDB analysis on PDB ID 6M3M (Positions 41–174).

| **No.** | **Residues** | **Number of residues** | **Score** |
| --- | --- | --- | --- |
| 1 | A:T92, A:R93, A:R94, A:I95, A:R96, A:M102, A:K103, A:D104, A:L105, A:S106, A:P107 | 11 | 0.773 |
| 2 | A:N49, A:T50, A:A51, A:T149, A:R150, A:N151, A:P152, A:A153, A:N154, A:N155, A:A156, A:A157 | 12 | 0.694 |
| 3 | A:N76, A:T77, A:N78, A:S79, A:S80, A:P81, A:D82, A:Q84, A:L114, A:G115, A:T116, A:G117, A:E119, A:A120, A:G121, A:L122, A:P123, A:Y124, A:G125, A:A126, A:N127, A:K128, A:D129, A:G130, A:T136, A:E137, A:G138, A:A139, A:L140, A:N141, A:T142, A:P143, A:K144, A:D145, A:H146, A:I147 | 36 | 0.562 |
| 4 | A:T55, A:H60, A:G61, A:K62, A:E63, A:D64, A:F67, A:P68, A:R69, A:G70, A:Q71, A:I75, A:I158, A:V159, A:L160, A:Q161, A:L162, A:P163, A:Q164, A:G165, A:T166, A:T167, A:L168, A:P169, A:K170, A:G171, A:Y173 | 27 | 0.561 |
