# Supplementary material for: In Silico Design of a Trans-Amplifying RNA-Based Vaccine against SARS-CoV-2 Structural Proteins
Source: Adv Virol. 2024 Sep 30;2024:3418062. doi: 10.1155/2024/3418062 (PMC11459942; doi:10.1155/2024/3418062)
Supplement: Supplementary Materials — Supplementary Tables 1, 2, 3, and 4: Predicted discontinuous B-cell epitopes of the Spike, Membrane, Nucleocapsid, and Envelope proteins, respectively, using ElliPro-IEDB analysis. Supplementary Table 5: Variants associated with the selected epitope-rich fragments. Supplementary Figure 1: Population coverage of the selected epitopes. [file 3418062.f1.zip › Supplementary Table 4.docx]

Supplementary Table 4. Predicted discontinuous B-cell epitopes of the Envelop protein using ElliPro-IEDB analysis on PDB ID 7K3G (Positions 8-38).

| **No.** | **Residues** | **Number of residues** | **Score** |
| --- | --- | --- | --- |
| 1 | A:L31, A:L34, A:T35, A:A36, A:L37, A:R38 | 6 | 0.769 |
| 2 | A:E8, A:T9, A:G10, A:T11, A:L12 | 5 | 0.613 |
