# Supplementary material for: In Silico Design of a Trans-Amplifying RNA-Based Vaccine against SARS-CoV-2 Structural Proteins
Source: Adv Virol. 2024 Sep 30;2024:3418062. doi: 10.1155/2024/3418062 (PMC11459942; doi:10.1155/2024/3418062)
Supplement: Supplementary Materials — Supplementary Tables 1, 2, 3, and 4: Predicted discontinuous B-cell epitopes of the Spike, Membrane, Nucleocapsid, and Envelope proteins, respectively, using ElliPro-IEDB analysis. Supplementary Table 5: Variants associated with the selected epitope-rich fragments. Supplementary Figure 1: Population coverage of the selected epitopes. [file 3418062.f1.zip › Supplementary Table 5.docx]

Supplementary Table 5. Variants associated with selected epitope-rich fragments from the S, N, E, and M proteins, with accession numbers P0DTC2, P0DTC9, P0DTC4, and P0DTC5, respectively. This information is manually curated and maintained by the UniProt database (<https://www.uniprot.org/>).

| **Fragment** | **Strains** | **Mutation** | **Status** | **Description** |
| --- | --- | --- | --- | --- |
| Fragment S1  Position:  51-75 | Omicron/EG.5.1 | Q52H | Curated |  |
|  | Eta/B.1.525, Omicron/BA.1 | A67V | Curated |  |
|  | Alpha/B.1.1.7, Eta/B.1.525, 19B/501T, Omicron/BA.1, Omicron/BA.4, Omicron/BA.5, Omicron/BQ.1.1 | ΔH69/V70 | Published  [1] | May compensate for reduced infectivity of RBD escape mutants, and increased incorporation of cleaved spike into virions |
|  | Lambda/C.37 | G75V | Curated |  |
| Fragment S2  Position: 812-835 | N/A | N/A | N/A | N/A |
| Fragment S3  Position: 339-359 | Omicron/BA.1, Omicron/BA.2, Omicron/BA.2.12.1, Omicron/BA.4, Omicron/BA.5, Omicron/BQ.1.1 | G339D | Curated |  |
|  | Omicron/BA.2.75, Omicron/XBB.1.5, Omicron/EG.5.1 | G339H | Curated |  |
|  | Mu/B.1.621 | R346K | Curated |  |
| Fragment S4  Position: 451-477 | Omicron/BA.2.12.1 | L452Q | Curated |  |
|  | Delta/B.1.617.2, Epsilon/B.1.427/B.1.429, Kappa/B.1.617.1, Lambda/C.37, 19B/501Y, Omicron/BA.4, Omicron/BA.5, Omicron/BQ.1.1 | L452R | Published  [2] | Contributes to cellular immunity evasion and increases infectivity |
|  | B.1.1.2981 | Y453F |  |  |
|  | Omicron/EG.5.1 | F456L | Curated |  |
|  | Omicron/BA.2.75, Omicron/BQ.1.1, Omicron/XBB.1.5, Omicron/EG.5.1 | N460K | Curated |  |
|  | 20A.EU2, Iota/B.1.526, Omicron/BA.1, Omicron/BA.2, Omicron/BA.2.12.1, Omicron/BA.2.75, Omicron/BA.4, Omicron/BA.5, Omicron/BQ.1.1, Omicron/XBB.1.5, Omicron/EG.5.1 | S477N | Curated |  |
| Fragment N2  Position: 151-174 | Omicron/BA.4 | P151S | Curated |  |
| Fragment E  Position: 1-17 | Omicron/BA.1, Omicron/BA.2, Omicron/BA.2.12.1, Omicron/BA.2.75, Omicron/BA.4, Omicron/BA.5, Omicron/BQ.1.1, Omicron/XBB.1.5 | T9I | Curated |  |
|  | Omicron/XBB.1.5, Omicron/EG.5.1 | T11A | Curated |  |
| Fragment M  Position: 1-20 | Omicron/BA.1 | D3G | Curated |  |
|  | Omicron/BA.5, Omicron/BQ.1.1 | D3N | Curated |  |
|  | Omicron/BA.1, Omicron/BA.2, Omicron/BA.2.12.1, Omicron/BA.2.75, Omicron/BA.4, Omicron/BA.5, Omicron/BQ.1.1, Omicron/XBB.1.5, Omicron/EG.5.1 | Q19E | Curated |  |

References:

1 Meng B, Kemp SA, Papa G, Datir R, Ferreira IA, Marelli S, et al. Recurrent emergence of SARS-CoV-2 spike deletion H69/V70 and its role in the Alpha variant B. 1.1. 7. Cell reports. (2021);**35**(13).

2 Motozono C, Toyoda M, Zahradnik J, Saito A, Nasser H, Tan TS, et al. SARS-CoV-2 spike L452R variant evades cellular immunity and increases infectivity. Cell host & microbe. (2021);**29**(7):1124-36. e11.
